# *EWSR1*—The Most Common Rearranged Gene in Soft Tissue Lesions, Which Also Occurs in Different Bone Lesions: An Updated Review

**DOI:** 10.3390/diagnostics11061093

**Published:** 2021-06-15

**Authors:** Uta Flucke, Max M. van Noesel, Vasiliki Siozopoulou, David Creytens, Bastiaan B. J. Tops, Joost M. van Gorp, Laura S. Hiemcke-Jiwa

**Affiliations:** 1 Department of Pathology, Radboud University Medical Center, 6525 GA Nijmegen, The Netherlands; 2 Princess Máxima Center for Pediatric Oncology, 3584 CS Utrecht, The Netherlands; M.M.vanNoesel@prinsesmaximacentrum.nl (M.M.v.N.); b.b.j.tops@prinsesmaximacentrum.nl (B.B.J.T.); l.s.jiwa-3@prinsesmaximacentrum.nl (L.S.H.-J.); 3 Division Cancer & Imaging, University Medical Center Utrecht, 3584 CX Utrecht, The Netherlands; 4 Department of Pathology, Antwerp University Hospital, 2650 Edegem, Belgium; vasiliki.siozopoulou@uza.be; 5 Department of Pathology, Ghent University Hospital, Ghent University, 9000 Ghent, Belgium; David.creytens@uzgent.be; 6 Department of Pathology, St Antonius Hospital, 3435 CM Nieuwegein, The Netherlands; j.van.gorp@antoniusziekenhuis.nl

**Keywords:** EWSR1, soft tissue tumors, bone tumors, pathology, molecular

## Abstract

EWSR1 belongs to the FET family of RNA-binding proteins including also Fused in Sarcoma (FUS), and TATA-box binding protein Associated Factor 15 (TAF15). As consequence of the multifunctional role of *EWSR1* leading to a high frequency of transcription of the chromosomal region where the gene is located, *EWSR1* is exposed to aberrations such as rearrangements. Consecutive binding to other genes leads to chimeric proteins inducing oncogenesis. The other TET family members are homologous. With the advent of widely used modern molecular techniques during the last decades, it has become obvious that *EWSR1* is involved in the development of diverse benign and malignant tumors with mesenchymal, neuroectodermal, and epithelial/myoepithelial features. As oncogenic transformation mediated by EWSR1-fusion proteins leads to such diverse tumor types, there must be a selection on the multipotent stem cell level. In this review, we will focus on the wide variety of soft tissue and bone entities, including benign and malignant lesions, harboring *EWSR1* rearrangement. Fusion gene analysis is the diagnostic gold standard in most of these tumors. We present clinicopathologic, immunohistochemical, and molecular features and discuss differential diagnoses.

## 1. Introduction

Ewing sarcoma was molecularly defined by Delattre et al. in 1992 upon the identification of the Ewing sarcoma breakpoint region 1 (*EWSR1*) located on chromosome 22q12.2 and the term for this gene was coined [1]. EWSR1 is a multifunctional protein ubiquitously expressed in most cell types, indicating diverse roles in physiological cellular processes, including organ development and aging. Genetic and epigenetic pathways are modulated by EWSR1 but the exact mechanisms are still poorly understood [2].

EWSR1 belongs to the FET (also known as TET) family of RNA-binding proteins that also includes Fused in Sarcoma (FUS), and TATA-box binding protein Associated Factor 15 (TAF15) [2]. As a consequence of the multifunctional role of *EWSR1* leading to a high frequency of transcription of the chromosomal region where the gene is located, *EWSR1* is exposed to aberrations such as rearrangements. Consecutive binding to other genes leads to chimeric proteins inducing oncogenesis. These various somatic genetic rearrangements involving *EWSR1* result in a fusion of its N-terminal coding region to the C-terminal DNA binding domain of one of several transcription factors. They are reported to act as aberrant transcription factors with the N-terminal domain of *EWSR1* as a strong transactivator. The other TET family members are homologous and are involved in strikingly similar translocation events giving rise to the production of structurally similar oncoproteins [3,4].

With the advent of widely used modern molecular techniques during the last decades, it has become obvious that *EWSR1* is involved in development of diverse benign and malignant tumors with mesenchymal, neuroectodermal, and epithelial/myoepithelial features [5]. As oncogenic transformation mediated by EWSR1-fusion proteins leads to such diverse tumor types, there must be a selection on a multipotent stem cell level [2].

In this review, we will focus on the wide variety of soft tissue and bone entities, including benign and malignant lesions, harboring *EWSR1* rearrangement. Fusion gene analysis is the diagnostic gold standard in most of these tumors. We present clinicopathologic, immunohistochemical, and molecular features and discuss differential diagnoses.

## 2. Ewing Sarcoma

Arthur Purdy Stout and James Ewing were the first to describe this aggressive small, blue round-cell entity in 1918 and 1921, respectively [6,7,8]. Later on, the chromosomal translocation (11;22) was found by Aurias et al. and Turc-Carel et al. in 1983, the second breakthrough of translocation/fusion-gene associated sarcomas following alveolar rhabdomyosarcoma (ARMS) [9,10,11]. Subsequently, the fusion gene has been detected as mentioned in the introduction [1], being the genetic hallmark by an otherwise aspecific small blue, round-cell tumor.

Ewing sarcoma, the prototypic round-cell sarcoma, is relatively common in comparison to other small blue round-cell sarcomas. It arises in soft tissue and bone of children, adolescents, and young adults. Exceptionally, older patients are affected. The mean age is in the second to third decade. White males have the highest incidence and black females the lowest due to ethnic genetic preposition differences. Tumors can originate anywhere in the body, and around 80% of the neoplasms arise in the bone with preference sites in decreasing order of frequency: lower extremities, pelvis, upper extremities, ribs, spine, and craniofacial. Distribution in the soft tissue is extremities, chest wall, retroperitoneum, paravertebral, pelvis, and head and neck. Visceral organs, skin, and epidural spaces are rarely involved [12,13]. The origin of the peripheral nerve as reported by Stout in 1918 can clinically be confused with malignant peripheral nerve sheath tumor [7].

Macroscopically, these infiltrative lesions are (multi)nodular), fleshy, and often necrotic. A pseudocapsule can be present in soft tissue neoplasms. Post-therapy specimens show fibrosis, necrosis, and hemorrhage, often without visible tumor [12,13].

**Figure 1:**
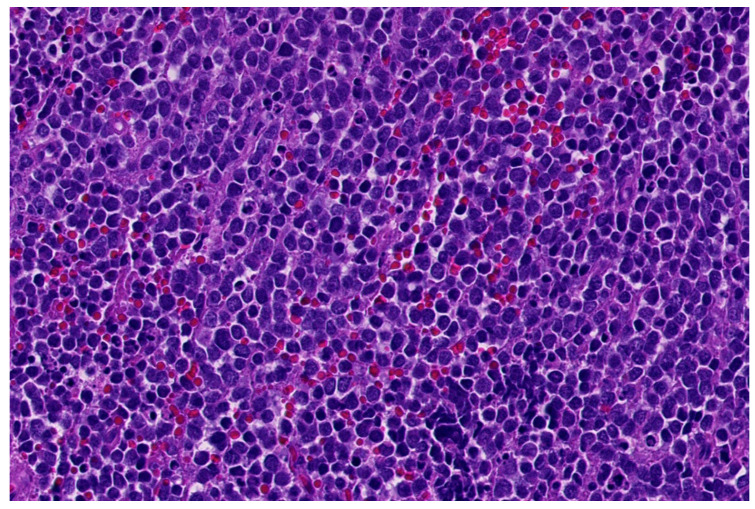
Classical morphology of Ewing sarcoma (HE; 40× magnification).

Histologically, Ewing sarcoma is composed of cellular sheets of relatively featureless small cells with round dark nuclei and inconspicuous cytoplasm (Figure 1). In some cases, cells are larger displaying more nuclear variability. The cytoplasm can appear clear due to retraction artefacts. Homer-Wright rosettes may be numerous in a subset of cases initially called peripheral primitive neuroectodermal tumors [6,13]. Adamantinoma-like Ewing sarcoma shows more cohesive sheets and nests of cells with peripheral palisading, prominent desmoplastic stroma with production of hyaline membrane collagen, presence of keratin pearl formation, and comedo-like necrosis. These lesions are predestinated for misinterpretation as carcinoma, since keratins, including high molecular keratins, p40, and p63, are commonly positive [6,14].

Immunohistochemically, CD99 is specific in its distinct staining pattern of the cell-membrane. Nuclear FLI and ERG expression is commonly observed in the cases with corresponding fusion genes. Neuroendocrine markers may be expressed. Keratin-expression, often dot-like, was found in 1/3 of the cases; it can be confused with small-cell carcinoma, especially when combined with the expression of p40 and p63 [6,14]. This is of particular importance in the head and neck area [14]. Expression of NKX2-2 in Ewing sarcoma seems to be highly sensitive, with imperfect specificity in comparison to other small, blue round-cell tumors [15,16,17,18,19]. Expression of desmin is reported in a few cases, and can be confused with ARMS or desmoplastic small round-cell tumor (DSRCT) [6,20].

Ewing sarcoma is genetically characterized by binding of *EWSR1* or other members of the TET/FET family to members of the ETS family [5]. Approximately 85–90% of the Ewing’s sarcomas display the translocation t(11;22)(q24;q12) resulting in the *EWS/FLI1* fusion gene, and approximately 5–10% harbor a *EWSR1-ERG* fusion gene [6]. The remaining cases show rare gene partners, such as *ETV1*, *ETV4*, and *FEV*, and *EWSR1* can be substituted by *FUS* [21].

Although prognosis has improved markedly for patients with primary disease (5-year survival rate around 65%), presence of metastatic disease at time of diagnosis or early relapse leads to an adverse prognosis (5-year survival rate around 25–30%), with adequate surgical resection, aggressive multimodal chemotherapy, and adjuvant local radiotherapy being the optimal treatments.

Differential diagnoses are listed in Table 1.

## 3. Round-Cell Sarcomas with *EWSR1*-*Non-ETS* Fusions

### 3.1. NFATc2-Rearranged Sarcoma

NFATc2-rearranged sarcoma was first described in 2009 by Szuhai et al. [22]. It is another, apparently very rare, primitive round-cell tumor with a methylation profile distinct from Ewing sarcoma, probably due to the non-ETS fusion gene [23]. Therefore, it belongs in the current WHO classification to the category of “round-cell sarcoma with *EWSR1*-non-ETS fusions” [5,24].

NFATc2-rearranged sarcoma affects males and females with predominance of males at least in the first studies. There is a broad age range from childhood to the elderly with a median age in the late third decade of life [24,25,26].

As Ewing sarcomas, these neoplasms originate mainly in the bone with predilection for the long bones, particularly femur and humerus. A few cases were reported to be localized in soft tissue and intraabdominally [25,27].

Histology demonstrates sheets, lobules, cords, and trabeculae of small, blue, round cells or less commonly spindle cells with slightly irregular nuclei. Nuclear pleomorphism is described in some lesions. There is a variable stromal reaction, being sclerotic, hyaline, myxoid, myxohyaline, and chondromyxoid. Cartilaginous or osteoid-like areas are rarely described [27].

Immunohistochemistry shows reaction for CD99; for AGGRECAN; and inconsistently for panCK AE, S100, BCOR, WT1, ERG, and ETV-4. Desmin, NKX3-1, and SATB2 are negative [27].

Amplification of the *EWSR1-NFATC* fusion gene is typical and can support the diagnosis when present [22,23,25,27].

In a few cases, rearranged *EWSR1* is substituted by *FUS* [26,27]. However, such cases show a different transcriptomic profile. While *EWSR1-NFATC* tumors were strongly enriched in genes associated with inflammatory response, the *FUS-NFATC2* tumors showed a signature of proliferation and drug resistance [26].

The outcome of patients is uncertain, since response to chemotherapy in unclear. Histological response to multimodal therapy seen in the resection-specimens was poor [27]. Patient studies until now have been too small to draw definitive conclusions for prognosis. Favorable outcome is reported in few cases with long-term follow-up, mostly after complete resection. No data on radiotherapy effect are available [25].

Differential diagnoses are listed in Table 2.

### 3.2. EWSR1-PATZ and EWSR1-VEZF1 Rearranged Sarcoma

*EWSR1-PATZ*-rearranged sarcomas were first reported by Mastrangelo et al. in 2000 [28]. Recently, more knowledge was gained based on advanced molecular technologies. *EWSR1-PATZ*-rearranged sarcoma seems to be a separate entity with a wide clinicopathological spectrum [26,29]. *VEZF1* is considered a paralogue of *PATZ*, and the few cases described with *EWSR1-VEZF1* are similar to the *PATZ*-rearranged cases in terms of morphology and immunoprofile [29,30,31].

The age ranges from early childhood to the elderly, with an average age in the fourth decade. The anatomic sites vary, with lesions being located superficially and deep, mainly in the trunk (thorax, including lung; abdomen), and rarely in the head and neck and extremities [29]. Intracranial localization is also reported [29,32].

Grossly, tumors are either well-circumscribed or infiltrating showing on the cut-surface a tan-yellow or grey-white color.

**Figure 2:**
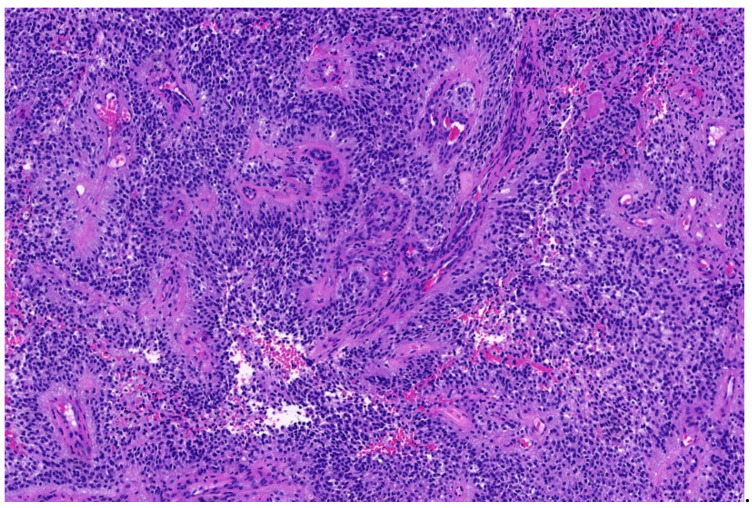
*EWSR1*-PATZ sarcoma with small blue round-cell morphology (HE; 20× magnification).

**Figure 3:**
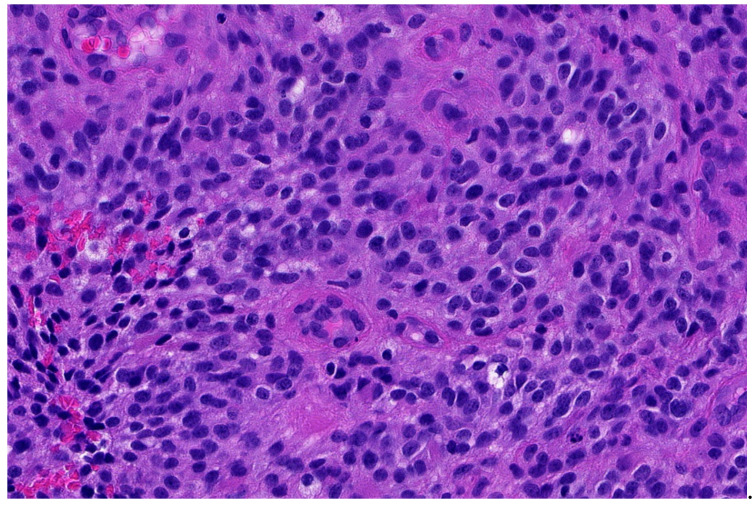
Higher magnification shows more irregular nuclei of the *EWSR1*-PATZ sarcoma in comparison to Ewing sarcoma (HE; 40× magnification).

Histology is striking variable ranging from small blue round-cell morphology (Figure 2 and Figure 3) to lesions showing a mixture of spindled, epithelioid, rhabdoid, ovoid, and round cells in varying fractions. The cells are commonly bland-looking with monomorphic nuclei and a low mitotic rate. They can be arranged in sheets, nests, and/or fascicles. Glandular structures as seen in synovial sarcoma, and pseudocystic spaces may be present. The stromal reaction is diverse and can be colleagenous, hyaline, or myxoid. There are often thick bands of collagen. Hyalinization of vessel walls are other possible features. Necrosis may occur but is mostly not prominent [29,33,34,35].

Michal et al. divided the tumors into two subgroups:

1. Low-grade appearing tumors: the spindled, epithelioid, ovoid, and round cells are set in a hyaline stroma reminiscent of solitary fibrous tumors and myoepithelioma.
2. Intermediate and high-grade appearing neoplasms have a round and/or ovoid morphology with few spindle cells and slight stromal component. The tumors of this second subgroup resemble other small, blue round-cell tumors, e.g., ARMS, BCOR-, and CIC-rearranged sarcoma.

Whether a transition may occur from low-grade to high-grade morphology as seen in myxoid/round-cell liposarcoma amongst others is yet unknown [29].

There is a polytypic immunophenotype with variable expression of neural, skeletal muscle, and epithelial markers. OLIG2 is also positive [29].

In contrast to NGS (or RT-PCR), FISH is not the method of choice for confirming the presence of the fusion gene due to the close proximity of the gene loci of *PATZ* and *EWSR1* on chromosome 22q12 [29,34].

Based on whole transcriptome sequencing, *EWSR1-PATZ* rearranged sarcomas are different from other *EWSR1*-related sarcomas [26].

Lesions can be aggressive or follow a more favorable course [29,33,34,35]. It is unclear if the above-mentioned morphological grading is prognostic for outcome. In addition, deletions of *CDKN2A/B* and *MDM2* gene amplification were associated with fatal outcome in one study and may therefore be a negative predictor of outcome [33].

### 3.3. Small Blue Round-Cell Tumor with EWSR1-SMARCA5 Rearrangement

A single case has been described in a 5-year-old female with a mass in the lumbosacral spinal canal with small blue round-cell histology and an immunoprofile like Ewing sarcoma. Cytogenetics showed a t(4;22) (q31;q22) as sole abnormality resulting in an *EWSR1-SMARCA5* fusion [36]. More cases are necessary for definitive categorization.

### 3.4. Desmoplastic Small Round-Cell Tumor (DSCRT)

The first description has been done by Gerald et al. in 1991, and one year later, the consistent translocation t(11;22)(p13;q12) was found by Sawyer et al. [37,38,39]. Ladanyi et al. detected the corresponding fusion gene *EWSR1-WT1* [40]. *WT1*, a suppressor of transcription, is expressed in primitive, developing mesothelium [41]. It is therefore not surprising that DSRCTs are classically located in the abdominal cavity with growth along the mesothelial membrane, often with multifocal spread at diagnosis. Origin in the small pelvis with ovary, or spermatic cord/paratesticular (tunica vaginalis), thoracic cavity/pleura, head and neck region, cranium/intracerebral, cauda equina, and extremities is rarely reported. Mostly adolescents and young adult males with a mean age in the second decade of life are affected, whereas females and older patients are rarely involved [21,38,41,42,43].

Grossly, this tumor is firm and shows a multinodular growth pattern with infiltration into adjacent structures and organs (e.g., liver). It has a solid and gray-white appearance with possible necrotic areas and hemorrhage [38,41].

**Figure 4:**
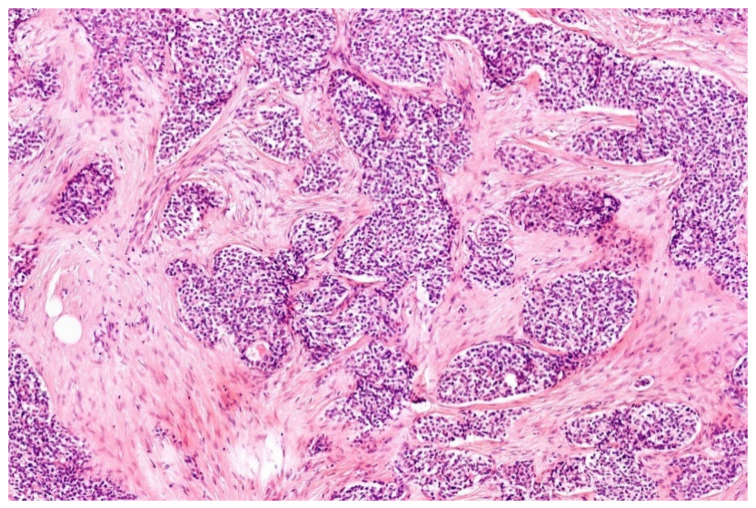
Small, blue, round cells situated in a desmoplastic stroma is characteristic for DSRCT (HE; 20× magnification).

Histologically, DSRCT is composed of irregular sheets, nests, trabecula, and cords of small cells with hyperchromatic nuclei and inconspicuous cytoplasm surrounded by a prominent desmoplastic stroma, which is a hallmark (Figure 4). When absent, other small round (or spindle) cell tumors could be superior in the differential diagnosis. Cells may vary in shape and size. The nuclei are round, ovoid, or spindly. In some cases, a rhabdoid/eosinophil or clear cytoplasm was noticed. The latter could be due to retraction during fixation process. Glandular/tubular or rosette-like structures have been identified in some cases. Mitotic figures may be numerous. Necrosis, possibly with calcification, can be present. The cells show immunohistochemically a polyphenotypic profile with expression of epithelial (EMA, broad-spectrum keratins (sometimes dot-like), myogenic (desmin, SMA in few cases), and neural markers (CD57, NSE) [21,38,41,43,44,45]. However, when this polyphenotype is incomplete, as sometimes seen, other small, blue round-cell tumors are conceivable, at least without the classical clinical context [46]. WT1 is only of diagnostic value when an antibody toward the carboxy terminus is used showing a nuclear staining pattern [21,43,47]. The presence of *EWSR1-WT1* confirms the diagnosis in >95% of the cases [41,43]. Multiple copies of the fusion gene have also been found [48].

The differential diagnoses besides other small blue round-cell sarcomas (Table 1 and Table 2) can be carcinoids and small-cell carcinoma, including Merkel cell carcinoma because of the desmoplastic stroma reaction [38,41]. Neuroblastoma, lymphoma, and blastemic Wilms tumor are probably less relevant in the differential-diagnostic spectrum [41] (see Table 3).

Treatment is challenging, and survival is low despite initial response to multimodal chemotherapy. Debulking is usually the surgical procedure, and tumor-free margins can often not be achieved when extended surgery is performed. For local control, several different additions have been tried with varying effects, such as total abdominal irradiation or surgery in combination with HIPEC. However, most patients relapse with locally disseminated disease not responsive to further treatment. Distant metastases may occur with involvement of lungs, liver, lymph nodes, bone, kidney, pancreas, spleen, adrenal gland, and small pelvis [41,43,44,49]. An indolent clinical course is reported in some mainly unusual cases [50,51].

## 4. Myxoid Liposarcoma (MLS)

MLS is the only translocation-associated liposarcoma-subtype recapitulating more or less normal lipogenesis with maturation arrest [5,52,53,54]. Limon et al. detected the most common translocation (12;16) in 1986 [55]. The specific fusion genes *FUS-DDIT3* (ca 90% of the cases) and, more rarely, *EWSR1-DDIT3* (in up to 10% of cases) are the result of the t(12;16)(q13;p11) and t(12;22)(q13;q12), respectively [56,57]. DDIT is an enhancer binding family of transcription factor involved in erythropoiesis and adipogenesis [58,59].

MLS is the most common liposarcoma arising in children, adolescents, and young adults [60,61,62,63]. It comprises up to 35% of all liposarcomas and has, in 1/3 of cases, the tendency to metastasize to other soft tissue sites, including mediastinum and retroperitoneum, and also to bone, lung, and liver, with a consecutive fatal outcome. The classical primary localization (2/3 of the cases) is the deep soft tissues commonly of the thigh [60,64]. Cases of the retroperitoneum are almost exclusively metastases, with some exceptions [65,66]. At distal sites of the extremities, this tumor is exceedingly rare [54].

Macroscopically, the lesion is multinodular and gelatinous on cut surface. High grade areas are firm and grey-white in appearance.

**Figure 5:**
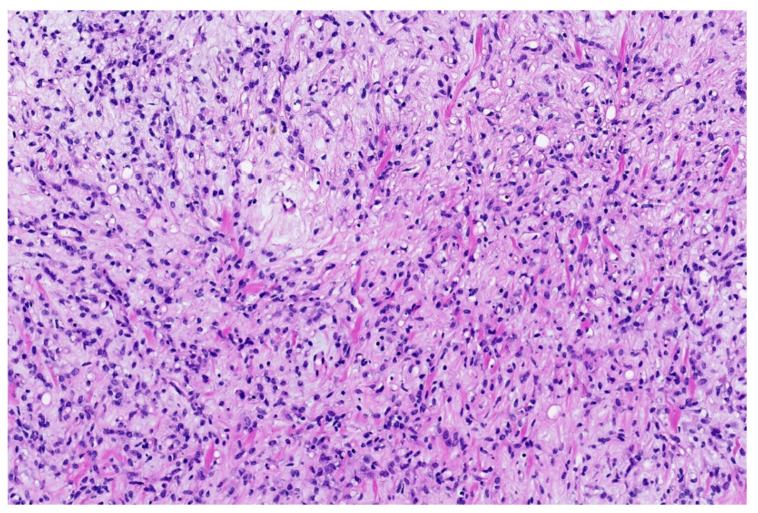
Myxoid liposarcoma showing different stages of primitive fat cells. There is slight pleomorphism. The myxoid matrix contains variable collagen (HE; 20× magnification).

Histology depicts a nodular growth pattern of relatively low cellularity with enhancement of cells at the periphery of the nodules. There is a proliferation of uniform bland, round-to-oval-shaped primitive cells intermingled with a variable amount of lipoblasts of different stages in an abundant myxoid stroma. Slightly pleomorphic nuclei are often associated with multivacuolated cytoplasm (Figure 5). In addition to the classical features, a nested pattern or islands of primitive cells, areas of extensive maturation, pseudoacini, or a cord-like growth pattern can be obvious. The very characteristic delicate plexiform (‘chicken-wire’) capillary vasculature is less obvious in cellular areas. When present, hemangiopericytoma-like vessels can be confused with solitary fibrous tumor, especially the lipomatous variant. Stromal hyalinization is rarely reported, which can be misleading if prominent. Hypercellular morphology with more large round cells with increased nuclear/cytoplasmic ratio, distinct nucleoli, and a small amount of intervening myxoid stroma is called round-cell liposarcoma and is associated with an inferior prognosis when more than 5% of the neoplasm is affected [5].

Immunohistochemistry shows variable expression of S100, which is of little value [21,54,62]. The determination of the fusion gene is therefore an important confirmation that has consequences therapeutically. Recently, nuclear expression of DDIT3 as an appropriate immunohistochemical surrogate marker has been reported [67,68,69].

In the differential diagnosis is lipoblastoma, which rarely occurs in adults, at least in its primitive form, and myxoid pleomorphic liposarcoma when slight pleomorphism is present. Chondroid lipoma can be considered because of lipoblastic differentiation, and soft tissue angiofibroma shows some similarities in terms of the branching capillaries, which do not have such a delicate appearance in the latter. Small, blue round-cell tumors can be pondered when round-cell morphology without obvious lipoblasts is observed [54] (see Table 1, Table 2 and Table 4).

Neo-adjuvant radiotherapy leading to a good response with maturation and hyalinization, followed by resection, is the optimal treatment [70]. Recurrences are less frequent. When metastasized (up to 1/3 of the patients), the outcome is poor; however, a slow progression may be observed.

## 5. Tumors with EWSR1/FUS Fused to the CREB-Family

ATF1, CREB1, and CREM are members of the CREB-family of transcription factors playing a pivotal role in diverse physiological processes [71,72]. They act as fusion partners of *EWSR1* or alternating *FUS* in several benign, intermediate, and fully malignant tumors, and the growing list includes mesenchymal, neuroectodermal, and epithelial neoplasms. Secondary genetic and/or epigenetic events seem to be mandatory for the specific oncogenesis. Whereas *EWSR1-ATF1* and *EWSR1-CREB* are the two most characterized fusions, *EWSR1-CREM* is less well studied [72]. Whether or not AFH (including its myxoid variant), PPMS, so-called mesothelioma, and *EWSR1-CREM* undifferentiated sarcoma are a spectrum of one tumor type will yield further studies in the future.

### 5.1. Angiomatoid Fibrous Histiocytoma (AFH)

This lesion was firstly recognized by Enzinger in 1979 with the main characteristics being reported in his seminal paper [73]. In 2000, the first genetic report was published showing *FUS-ATF1* as the result of t(12;16) (q13;p11) [74]. Later on, it became apparent that *EWSR1/FUS-CREB/ATF1/CREM* are the alternating candidates for the gene fusion, with *EWSR1-CREB1* being the most common in soft tissue lesions [21,75,76]. *EWSR1-CREM* positive cases are recently reported [72]. Multiple copy numbers of the fusion gene seem to be associated with pleomorphism [76].

AFH affect mainly children, adolescents, and young adults. However, the age range is broad. When located in superficial soft tissue, clinical presentation is often a palpable, slowly growing indolent nodus imposing as hemangioma or lymph node [21,73,77,78]. Rarely accompanied systemic symptoms such as malaise, pyrexia, and anemia are documented [21,78,79,80]. Classically, AFH arises subcutaneously in the extremities followed by the trunk and head and neck [21,73,77]. Involvement of deep soft tissues and visceral sites is increasingly reported due to higher diagnostic standards (including molecular diagnostics). They include mediastinum, retroperitoneum, intraabdominal, lung, brain, bone, and ovary [78,80,81,82,83].

Grossly, the neoplasm is circumscribed, (multi)nodular or (multi)cystic, grayish-yellow, and hemorrhagic, and can be as large as 10 cm [73,80].

**Figure 6:**
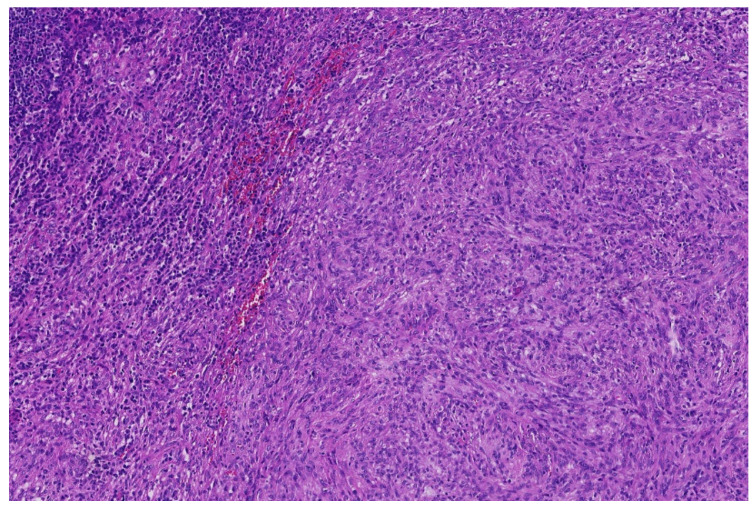
Angiomatoid fibrous histiocytoma showing sheets and short fascicles of histiocytoid cells with monomorphic nuclei. Note the lymphocytic reaction (HE; 20× magnification).

Histologically, these circumscribed (multi)nodular and/or multicystic lesions possess a fibrous pseudocapsule that is surrounded by a prominent often lymph node-like mixed lymphocytic infiltrate with variable germinal center formation and presence of plasma cells. The inflammatory component may also be intermixed with tumor. The tumor cells are arranged in sheets, nodules/whorls, aggregates, short fascicles (storiform), and reticular formations (when myxoid). The cells are histiocytoid with a syncytial aspect showing an ovoid, epithelioid, or spindled appearance and bland-looking nuclei with fine chromatin and moderate amount of ill-defined eosinophilic cytoplasm (Figure 6). Centrally, a cannonball arrangement can be seen. Unusual characteristics are scattered large cells, nuclear grooving, and bizarre and irregularly folded nuclei. Rhabdoid, clear cells, or osteoclast-like giant cells and Ewing-like areas may be present. Nuclear palisading as seen in schwannomas has been rarely observed. Mitotic rate is usually low, but atypical mitoses are not a worrisome sign. Hemorrhage and blood-filled spaces are, when present, a hallmark of this lesion often associated with hemosiderin deposition [21,73,77,78,79,80,81,82,84]. The stroma can be unremarkable, sclerotic, “desmoplastic”, or myxoid. Edema can be prominent, and a slit-like pseudovascular pattern may be seen. Additionally, perivascular hyalinization is a possible sign [80,82,84].

Immunohistochemistry shows expression of ALK in almost all cases and desmin in approximately 50% of the cases. EMA, CD99, and CD68 are variably expressed. Other smooth muscle markers such as SMA and caldesmon are sometimes positive. CD21 may be positive in some cells. Myogenin and MYOD1 are consistently absent [21,77,79,80].

Intracranial myxoid mesenchymal tumors are suggested to be a variant of AFH [83,85]. This meets the observations of peripherally located AFHs with myxoid changes [72,84,86]. Differential diagnoses are listed in Table 5.

AFH has a low to intermediate biologic potential, with most cases behaving benign. Recurrence is reported in up to 15% of the cases, especially when marginally excised. Metastases have been reported in up to 5%, most frequently in the regional lymph nodes and exceptionally in the lungs, liver, and brain. Pleomorphism and increased mitotic activity are not associated with worse outcome [21,77,82].

### 5.2. Primary Pulmonary Myxoid Sarcoma (PPMS)

PPMS, an entity rendered by Thway et al. in 2011, is an *EWSR1-CREB1* fusion gene associated neoplasm of the lung. It predominantly arises intrabronchially and involves the pulmonary parenchyma. It affects young to middle-aged adults, mainly women [21,87].

**Figure 7:**
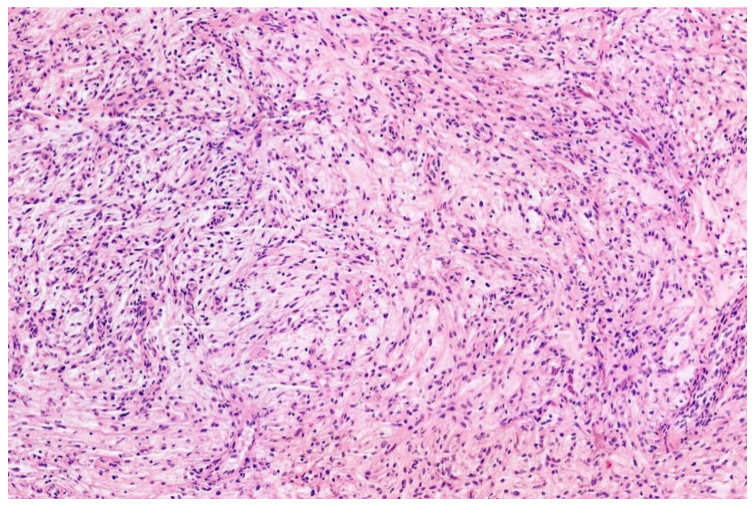
Primary pulmonary myxoid sarcoma: relatively uniform spindle cells arranged in loose fascicles in a myxoid matrix (HE; 20× magnification).

Morphologically, lesions are (multi)nodular with a pale and glistening cut surface on macroscopy. Microscopically, round-to-ovoid or spindle cells are situated in a myxoid matrix arranged in cords with possible reticular growth, as seen in extraskeletal myxoid chondrosarcoma (see there), nests, and sheets. Cells are relatively bland looking with at most slight nuclear pleomorphism (Figure 7). There is a low mitotic index and possibly focal necrosis. An inflammatory component is often obvious with presence of lymph follicles, either marginal or intralesional. Immunohistochemistry is of little value, with a faint reaction for EMA, but helps to exclude other lesions [21,87].

Lesions may be benign or malignant (with described metastases in kidney and brain) [21,87].

To what extent AFH and PPMS are related needs to be further explored. A close relationship is conceivable. Differential diagnoses are listed in Table 6.

### 5.3. EWSR1-CREM Undifferentiated Sarcoma

Currently, these tumors are still relatively unexplored.

One aggressive intraabdominal tumor in an adolescent was described consisting of swirls of uniform spindle cells with intercellular delicate collagen. Immunohistochemistry shows expression of vimentin, cytokeratin AE1/3, and CD56. EMA, CD34, ALK, synaptophysin, and DOG1 were focally positive. INI1 and H3K27me3 were retained. Negative were desmin, myogenin, S100, SOX10, NUT, CD31, and smooth muscle markers [72].

The other tumor was that of a 63-year-old woman with localization in the chest wall and a sclerosing epitheloid fibrosarcoma-like morphology with MUC4 and synaptophysin expression. The stroma was more fibrillary than sclerotic. After 17 months, there was no evidence of disease [72]. A comparable case was included in the study by Arbajian et al. 2017 [88,89]. Sequencing techniques should be applied in the right context to detect this rearrangement.

### 5.4. Clear-Cell Sarcoma (CCS)

Enzinger was the first to describe CCSs systematically in 1965 [90] (Enzinger 1965), also called melanoma of soft parts, because of overlapping morphological and immunohistochemical features with melanoma [91]. In the years 1990, 1991, and 1992, the translocation (12;22)(q13;q12) was found [92,93,94], one year after the *EWSR1-ATF1* gene fusion by Zucman et al. 1993 [95]. Later on, it became apparent that *CREB1* and *CREM* are substituents of *ATF1* involving a smaller subset of cases [72,96,97].

Young adults (30–40 years) with equal sex distribution are the main group of patients. However, there is a broad age range from children to the elderly [90,91,92,97,98,99,100,101,102]. Additionally, there is a race distribution with the overwhelming majority of patients being Caucasian, whereas black people and Asians are uncommonly afflicted [101].

The deep soft tissue of the extremities is for the most part involved. Superficial soft tissue and skin localization does not exclude diagnosis. Extension into bone can be seen. Unusual tumor sites include the trunk, e.g., breast, anus, mediastinum, pleura, retroperitoneum, and the head and neck area. Symptoms depend on site and are non-specific. Tumors can be large (up to 15 cm) [90,91,92,97,98,101,102,103,104].

Grossly, tumors are firm and roughly spherical, with a smooth, nodular, or coarsely lobular surface. Most are well defined and surrounded by a fibrous pseudocapsule. Ill-defined lesions are less frequently reported. The cut surface is usually gray to white. Brown areas and gelatinous foci are observed [90,102].

**Figure 8:**
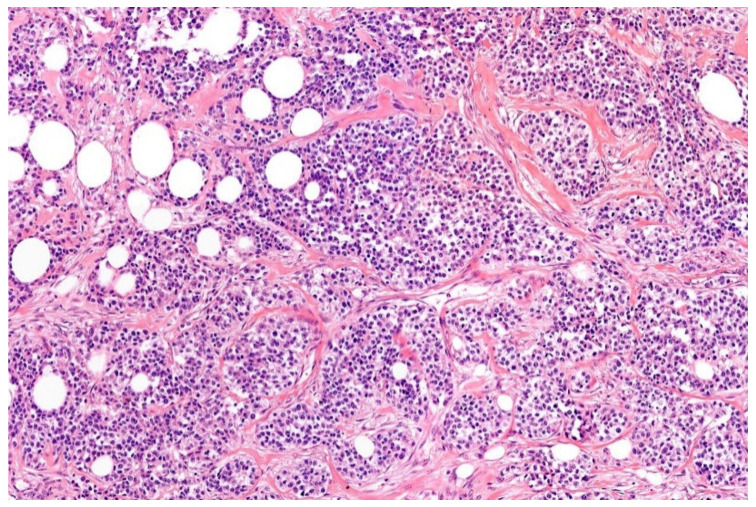
Clear-cell sarcoma comprises of nests of epithelioid cells separated by fibrous septa. There are uniform round nuclei and eosinophilic to clear cytoplasm (HE; 20× magnification).

Microscopically, the classical pattern is that of a well delineated tumor with extension into adjacent structures consisting of nests or short fascicles of monomorphic round-to spindled cells with ample clear to eosinophilic cytoplasm, vesicular nuclei, and prominent nucleoli (Figure 8). Mitotic activity is variable but rarely brisk. Cells are separated by delicate fibrous septa. Typical are multinucleated giant cells with wreath-shaped nuclei. A diffuse growth pattern and pleomorphism are seen in some instances. Also reported are rhabdoid cells, an alveolar growth pattern, a seminoma-like appearance with a lymphocyte-rich fibrovascular stroma, and a dominant stromal reaction. Melanin pigment may be present, and necrosis can be found [90,91,92,97,98,99,102,104].

Melanoma markers are expressed using immunohistochemistry [97,98,99,104].

Melanoma is the most important differential diagnosis. It has to be discriminate from CCS because of the therapeutic consequences [105]. Other differential diagnoses are listed in Table 7.

Excision is the treatment of choice. In a large epidemiological study, radiotherapy and chemotherapy were applied in 34% and 20%, respectively [101]. Whether this confers any survival advantage is unclear. At least some tumors are reported to be chemo-sensitive [99]. Local recurrences and in-transit metastases are reported in around 20% of the cases [99]. Sites of metastases are the lung (most commonly) and lymph nodes. The propensity to metastasize to lymph nodes is typical in comparison to most other sarcoma types. Overall, estimated 5- and 10-year survival is approximately 50% and 38%, respectively [98,99,101,102].

### 5.5. Clear-Cell Sarcoma-Like Tumor of the Gastrointestinal Tract (Osteoclastrich Tumor of the Gastrointestinal Tract or Malignant Gastrointestinal Neuroectodermal Tumor)

In 1993, Ekfors et al. described the first case of a clear-cell sarcoma in the duodenum [106]. However, a similar case was already reported in 1985 under the term malignant neuroendocrine tumor of the jejunum with osteoclast-like giant cells [107]. In 1998, it became apparent that these tumors share the same genetic characteristics [108], with most tumors harboring an *EWSR1-CREB1* fusion and less often an *EWSR1-ATF1* fusion [109]. Since then, there has been discussion whether these tumors are CCSs or a separate entity as they have morphological and genetic similarities [21,110,111,112].

Tumors arise in the gastrointestinal tract (small bowel, stomach, colon, and esophagus) of predominantly young adults and children. However, the age range is broad, including the elderly. An abdominal mass with pain and intestinal obstruction are the main clinical features. Lesions may be large, up to 15 cm. The appear macroscopically firm, solid, and tan-white. Microscopy shows primary involvement of submucosa and muscularis propria, occasionally with mucosal involvement [21,110,111,112]. Cytomorphology resembles CSS. However, tumors show, besides the nested pattern, arrangement in sheets. Pseudoalveolar, pseudopapillary, microcystic, fascicular, and cord-like patterns, and rosette-like structures, are also reported. Mitotic activity is variable. Osteoclast-like giant cells are often numerous [21,110,111,112]. Immunohistochemistry shows expression of S100 and SOX10. Other melanocytic markers (Melan A, HMB45) are in contrast to CSS commonly absent. Neural and neuroendocrine markers, including synaptophysin, NSE, and CD56, are inconsistently expressed. Rarely dot-like keratin-expression can be observed [21,110,111,112]. Although there are some differences compared to CCS with regard to morphology and immunophenotype, lack of melanin pigment does not exclude CCS [109]. Another important differential diagnosis regards melanoma. Although molecular alterations can often resolve this matter, not all melanomas harbor a *BRAF* mutation and not all clear-cell sarcoma-like tumors of the gastrointestinal tract have *EWSR1* rearrangements.

These tumors show aggressive behavior with metastases to lymph nodes and the liver [21,110,111,112].

### 5.6. Mesothelioma

**Figure 9:**
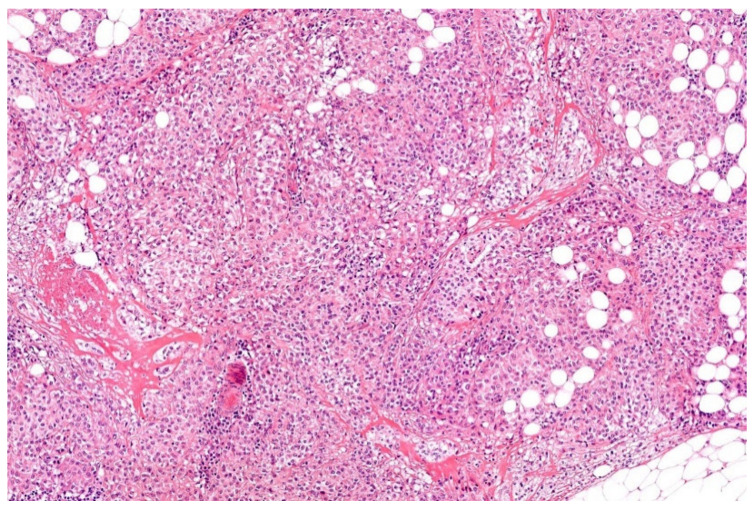
Mesothelioma consist sheets and nests of uniform epitheloid tumor cells with enlarged nuclei and eosinophilic cytoplasm (HE; 20× magnification).

In 2013 a (14;22)(q32;q12) translocation leading to a *EWSR1-YY1* fusion was reported in two mesotheliomas, showing for the first time fusion genes in these neoplasms [113]. In 2017, *EWSR1/FUS-CREB* fusions have been described in a subset of malignant mesotheliomas occurring mainly in young adults [114]. However, the age range is broad, comprising patients from the childhood to the elderly [113,114]. There is an equal sex distribution [115]. The peritoneum seems to be mostly involved with pleural lesions less frequently reported [114]. Extension into adjacent organs and structures and lymph node involvement are reported [115]. Histologically, these lesions resemble AFH and in part CSS as described above under these headlines (Figure 9). Additionally, papillary projections, acinar, and tubular structures and psammomatous calcifications are reported as seen in classical mesotheliomas. The cells are epithelioid and histiocytoid with monomorphic round-to-oval nuclei and eosinophilic cytoplasm [113,115]. The immunophenotype with keratin- and WT1 nuclear expression and absence of S100 differs from AFH and CSS. Overlapping positive immunohistochemical markers are EMA and desmin. Loss of BAP1 may occur in a minority of cases [113,115]. Other differential diagnoses are listed in Table 8. *EWSR1/FUS-ATF1/CREM* are the described fusion genes showing the spectrum seen in other entities with *EWSR1/FUS-CREB* [115]. Follow-up data show variable clinical presentation ranging from indolent to aggressive behavior [114,115].

## 6. Myoepithelial Tumors

Different from most other *EWSR1*-rearranged neoplasms, myoepithelial tumors have a normal counterpart, with myoepithelial cells being the outer layer of glands present in e.g., salivary glands, lung, skin adnexa, and mamma, but naturally not in soft tissue and bone.

The first myoepithelial tumor of soft tissue was published by Stout and Gorman in 1959 in a series of cutaneous lesions, and the first bone myoepithelioma was reported in 2001 [116,117]. Parachordoma is another term introduced 1977 in the English literature [118]. Reports of cytogenetic analyses showed heterogeneous abnormalities [119,120,121,122,123,124].

Since *EWSR1* rearrangement was mentioned in one myoepithelial carcinoma and one myoepithelioma of soft tissue 2007 and 2008 [125,126]; systematic analyses revealed that approximately 50% of myoepithelial tumors of skin, soft tissue, viscera, and bone harbor a *EWSR1* fusion gene with a variety of gene partners, including *PBX1*, *PBX3*, *ZNF 444*, *POU5F1*, *ATF1*, and *KLF17* [126,127,128,129,130,131,132]. *EWSR1* seems to be rarely substituted by *FUS* [128,129,130,132]. *PLAG1* rearrangement and other genetic changes are alternatively observed [124,133,134].

The age range is broad from early childhood to the elderly. Extremities and limb girdles are most frequently involved, followed by the head and neck and trunk. Skin, subcutis, and deep soft tissue, including mediastinum and retroperitoneum, can be affected [125,135,136,137]. Bone lesions most often arise in long tubular bones but also in small tubular bones and the axial skeleton, including iliac bone, sacrum, vertebra, ribs, skull, and jaw. Cortical destruction and extension into surrounding soft tissue may be present [138].

Macroscopically, tumors can be large with up to 20 cm. They are usually circumscribed and (multi)nodular. The cut surface often is white-grey in color with gelatinous areas. Calcification and ossification may be seen [137,138].

**Figure 10:**
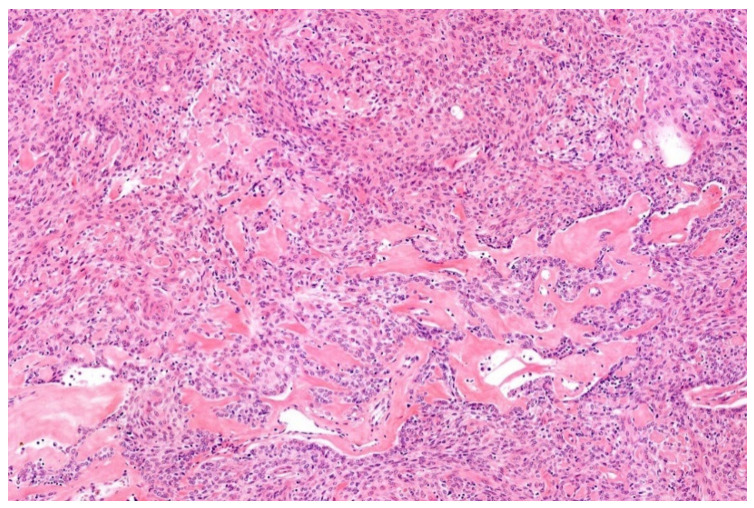
Myoepithelioma: epitheloid and spindle cells are arranged in sheets possessing bland looking nuclei. Note the prominent hyaline matrix (HE; 20× magnification).

Microscopy is similar to salivary gland myoepithelial tumors showing a (multi)nodular appearance with well-circumscribed nodi/noduli variably infiltrating adjacent tissue. There is a broad spectrum in terms of architecture, cellularity, and cell composition. Growth patterns, often combined, are solid, nested, reticular, trabecular, cord-like, and glandular. Cells are epithelioid and/or spindled, having sometimes a clear cytoplasm, and/or plasmacytoid, and/or rhabdoid (Figure 10) [135,136,137]. Lesions called syncytial myoepitheliomas mainly occurring in skin show a sheet-like syncytial growth of ovoid to spindled or histiocytoid cells with pale eosinophilic cytoplasm [139]. Criteria for malignancy were established in the largest series of soft tissue myoepithelial tumors, with tumors with benign cytomorphology or mild atypia (little variation in cell size, small relatively uniform nuclei, fine chromatin, inconspicuous nucleoli) diagnosed as myoepithelioma, whereas morderate to servere atypia (nuclear pleomorphism or vesicular or coarse chromatin, prominent nucleoli) represented features of myoepithelial carcinoma [135]. Small round-cell morphology has been described in myoepithelial carcinomas [125,136,137]. The matrix, variably present in myoepithelial tumors, can be (chondro)myxoid and/or collagenous/hyaline. Metaplastic cartilage or bone may occur. In myoepithelial carcinomas, malignant bone or cartilage can be observed. High mitotic rate and necrosis is reported in myoepithelial carcinomas [125,136,137].

The immunohistochemical profile of myoepithelial tumors is variable showing per definition expression of broad-spectrum keratins and/or EMA and neuronal markers as S100, SOX10, and/or GFAP. P63 is positive in a subset of cases. Smooth muscle markers (SMA, calponin, and desmin) are possibly positive. INI1 is lost in a subset of myoepithelial carcinoma [5]. MUC4 expression can be confusing when considering sclerosing epithelioid fibrosarcoma [130]. Nuclear expression of brachyury, absent in myoepithelial tumors, distinguishes them from chordomas [140]. Differential diagnoses are listed in Table 9.

Excision is the treatment of choice. Most of the lesions are superficially located with a benign morphology behaving indolent. Benign and malignant lesions have the potential for local recurrence. The metastatic rate of myoepithelial carcinoma is high with spread to lung, lymph nodes, bone, and soft tissue. Radiotherapy and chemotherapy are additional treatment options, but clinical effectiveness is variable [5,137].

## 7. Low-Grade Fibromyxoid Sarcoma (LGFMS)/Sclerosing Epithelioid Fibrosarcoma (SEF)

These mostly deep situated sarcomas show overlapping features in terms of morphology, immunohistochemistry, genetic aberrations, and behavior [141]. Therefore, it has been suggested that they form a spectrum of one entity [141,142].

LGFMS was firstly observed by Evans in 1987, and the first description of SEF was done by Meis-Kindblom eight years later [143,144]. The chromosomal translocation, most typical for LGFMS, (t7;16)(q34;p11), has been described in 2003 by Reid et al. and the corresponding fusion gene *FUS-CREB3L2* in the same year [145,146]. Later on, it was shown that *CREB3L1* is an alternative fusion partner of *FUS* and that *FUS* can be substituted by *EWSR1* [147,148]. The genetic findings of LGFMS were also found in SEF and hybrid cases with predominance of *EWSR1-CREBL3L1* in SEF [149]. In one case, a PAX5-CREB3L1 was identified [88].

Both entities affect patients over a wide age range with a median in the 3th (LGFMS) and 4th (SEF) decade [141,142,144,150]. Most often, these tumors occur in the deep soft tissue of the lower extremities, particularly thigh and limb girdles and the trunk. However, a wide variety of involved anatomic sites are reported, including intraabdominal, kidney, and bone [5,141,142,143,144,149,150,151,152,153].

Macroscopically, lesions are (multi)nodular with a grey-white whorled cut surface. Myxoid areas, if present, are visible. Infiltrative growth in adjacent structures can be seen [142,144,151].

**Figure 11:**
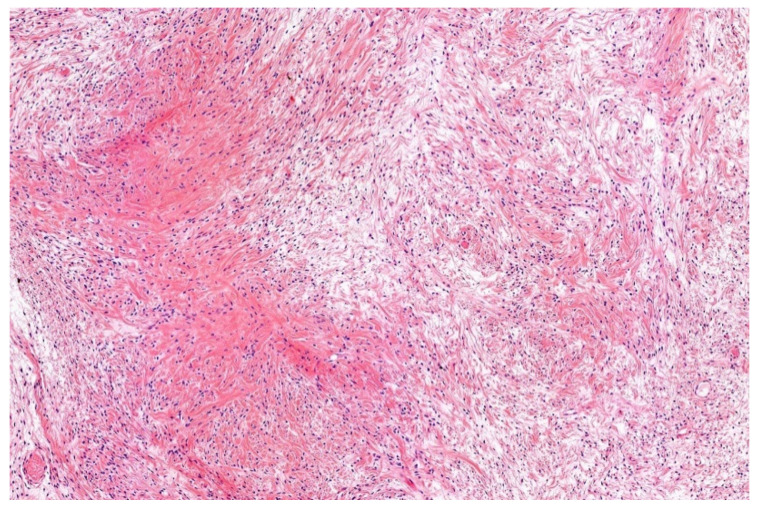
Low-grade fibromyoid sarcomas are characterized by bland looking spindle cells set in an alternating fibromyxoid matrix (HE; 20× magnification).

Microscopically, LGFMS is characterized by alternating fibrous and myxoid areas with a whorled and bundled growth of uniform bland-looking slender fibroblastic spindle cells with elongated and tapered nuclei. A storiform, fascicular, and patternless architecture may be seen (Figure 11). Mitotic figures are sparse. There is scant cytoplasm. Typically, there are arcades of small blood vessels. In some cases, hyaline rosettes surrounded by round or oval cells are present. Such neoplasms were formerly called hyalinizing spindle cell tumor with giant rosettes. Cellular examples containing epithelioid cells show overlap with SEF and hybrid cases occur. A shift of the LGFMS pattern to SEF morphology is described in recurrences and metastases. Uncommonly noted are cell clusters, strands, palisades, and a retiform pattern. Thick collagen bundles are sometimes found in fibrotic areas. Nuclear pleomorphism and multinucleated giant cells are rarely observed and are mainly associated with recurrences and metastases. Cystic changes and osseous metaplasia may occur [141,143,150,151].

**Figure 12:**
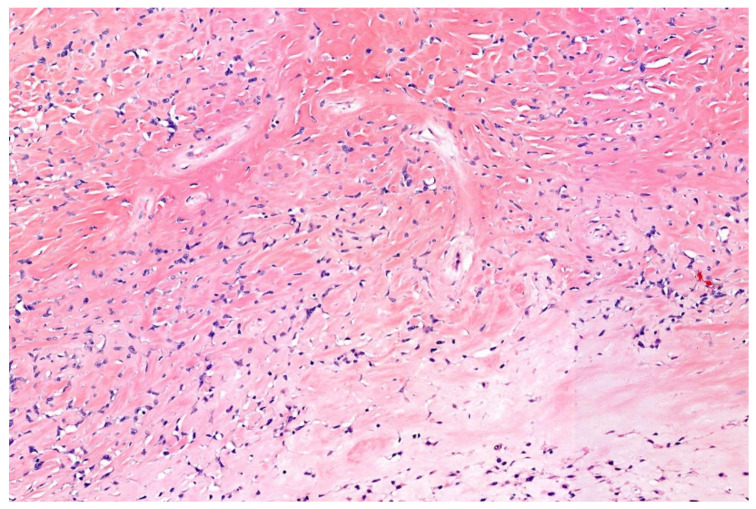
Sclerosing epitheloid fibrosarcoma, demonstrated by cords of bland looking epitheloid cells in a sclerotic stroma. Note pseudoangiomatous shrinkage artefacts (20× magnification).

SEF shows histomorphologically epithelioid/polygonal cells arranged in cords, nests, and sheets situated in a sclerotic stroma. Due to cellular shrinkage, a pseudovascular appearance can become apparent. The round-to-oval nuclei show at most slight pleomorphism and an open chromatin. There is a variable often low mitotic count (Figure 12) [141,142,144]. Chondro-osseous differentiation is exceptionally observed [144,149].

The most reliable immunohistochemical marker is MUC4 with positivity in the majority of LGFMS, whereas SEFs are positive in around 70% of the cases [149,154,155]. Other markers such as EMA, S100, CD34, SMA, and keratins (in SEF) are inconsistently expressed [141].

Differential diagnoses are listed in Table 10 and Table 11.

Wide excision is the treatment of choice. LGFMS typically shows a prolonged clinical course with recurrences and metastases. SEF seems to be more aggressive with much shorter survival; however, the outcome is variable [141,150].

## 8. Extraskeletal Myxoid Chondrosarcoma (EMC)

When initially described by Stout and Verner in 1953, it was thought that EMCs are true chondrosarcomas [156]. The first large series delineating this tumor type more precisely was published by Enzinger and Shiraki in 1972 [157]. In 1985, Hinrichs et al. reported for the first time the specific reciprocal translocation t(9;22)(q22;q11) leading to the most common fusion gene *EWSR1-NR4A3*, which was detected by Labelle et al. 1995 [158,159]. It seems that *NR4A3* is necessarily involved. The described fusion partners besides *EWSR1* are *TAF15*, *TCF12*, and *TFG* [160,161].

This in deep subcutis and soft tissue located sarcoma affects adults with a mean age of 50 years. Children are rarely involved [162,163]. Males are slightly more often affected [160]. The main sites are the proximal extremities and limb girdles followed by the distal extremities and trunk. Unusual sites are the head and neck area, including the intracranial cavity, the pelvic cavity/retroperitoneum, or intraabdominal and acral sites [21,160,162]. Rarely bone lesions are also reported [164].

Macroscopically, tumors show a (multi)nodular configuration with relatively well-defined margins and variably a pseudocapsule. The cut-surface appears gelatinous with a tan color. Firm grey-white areas and hemorrhage may be seen [160].

**Figure 13:**
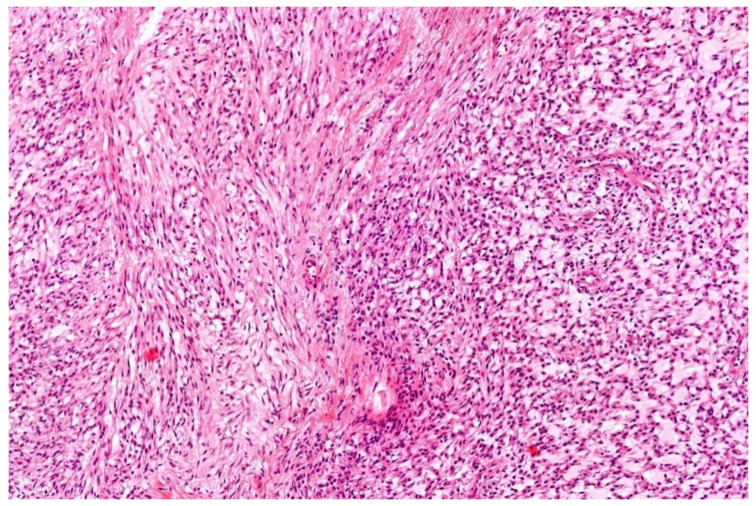
Extraskeletal myxoid chondrosarcoma shows a lace-like architecture due to the myxoid matrix. There are monomorphic epitheloid and spindle cells with an obvious eosinophilic cytoplasm (HE; 20× magnification).

Regarding microscopy, EMC is commonly a hypocellular lesion characterized by a multinodular growth pattern with presence of fibrous septa. The tumor nodules show peripheral accentuation of cellularity and are composed of bland-looking small round-to-spindled cells with scanty eosinophilic cytoplasm set in a myxoid matrix resulting in a lace-like or reticular appearance. The nuclei are usually uniform, round-to-oval with an open chromatin or with hyperchromasia (Figure 13). There is low mitotic activity. Cellular areas lose their classical architecture owing to the limited myxoid matrix. They may be present in primary and recurrent lesions sometimes associated with pleomorphism of epithelioid, rhabdoid, and spindled cells [160,162].

Immunohistochemistry is of little value depicting focal S100 reaction. GFAP, EMA, SMA, keratins, and p63 are expressed in a minority of cases with a focal staining pattern [160,161]. Fusion gene analyses is especially helpful when classical features are less obvious.

The most important differential diagnosis is myoepithelial tumors of soft tissue. They show morphological, immunophenotypical (EMA/Keratins + and S100/SOX10/GFAP+), and genetic overlap with rearrangement of *EWSR1* in a subset of cases. Fusion chimera involving *NR4A3* are confirmatory for the diagnosis of EMC [161]. For further differential diagnoses see Table 9.

Surgery is the treatment of choice. EMC shows a protracted clinical course with a high rate of recurrences and metastatic potential [21,162,165].

## 9. EWSR1-SMAD3-Positive Fibroblastic Tumor (ESFT)

This recently defined tumor type was first described by Kao et al. in 2018 [166]. Few reports have followed since then [167,168,169,170,171].

Lesions usually present as a relatively small painless mass in the skin and superficial soft tissue of the extremities, mainly distal, especially the foot. Occurrence in bone is reported in one case localized in the tibia [171]. There is a broad age range from the early childhood to the elderly and an obvious female preponderance.

Macroscopically, the neoplasms are nodular and firm, showing on cut surface a white-grey solid appearance.

**Figure 14:**
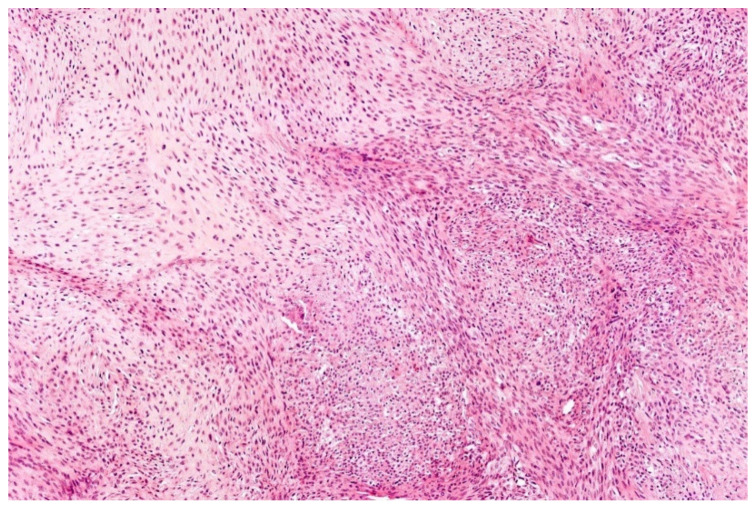
*EWSR1-SMAD3*-positive fibroblastic tumor is composed of fascicles of bland looking spindle cells. There is an alternating cellularity with a hyaline matrix (HE; 20× magnification).

Histologically, tumors have a nodular configuration and consist of infiltrative growing intersecting long or short fascicles. The spindle cells possess uniform elongated nuclei with open chromatin. Mitotic activity is low. There is inconspicuous cytoplasm. Cellular areas merge with hyalinized areas showing sometimes calcifications. In some cases, a zonation pattern is seen often with central hyalinization (Figure 14). When located intradermal, an epidermal collarette may be present. Arrangement around blood vessels as seen in myopericytomas is sometimes observed. Myxoid and collagenous areas with the latter reminiscent of collagen rosettes are rarely reported [167,168,169,170,171].

Immunohistochemically, the most reliable marker by now seems to be ERG demonstrating an homogeneous nuclear expression. Variable positive markers are SMA, keratins (both mostly week and focal), and SATB2. Reported negative stainings are EMA, desmin, S100, SOX10, CD34, CD31, MUC4, STAT6, TLE1, HMB45, and CD99. When a classical clinicopathologic constellation is present with expression of ERG, the diagnosis is straight forward. However fusion gene analysis may aid for the precise diagnosis, because benign and malignant lesions are in the differential diagnoses listed in Table 12 [167,168,169,170,171].

The clinical behavior appears to be benign, also when located in the bone, but lesions are prone to local recurrence even after 10 years [167,168,169,170,171].

## 10. Epithelioid and Spindle Cell Rhabdomyosarcoma with EWSR1/FUS-TFCP2 Fusion

These lesions were first described by Watson et al. in 2018 [26].

Hitherto-reported cases arose mainly in the bone and rarely in soft tissue, with predilection for the craniofacial bones. However, sites are heterogeneous, including also pelvis, femur, groin, and peritoneum. Intraosseous lesions show destruction of the cortex and expansion into soft tissue. The age range is broad, including pediatric patients and elderly patients. The average age is in the third decade. Males and females are affected (almost) equally with a slight female preponderance [172].

Macroscopically, a solid mass is reported [173].

Histologically, tumors consist of epithelioid and/or spindle cells. Whereas epithelioid cells are arranged in sheets, spindle cells show fascicular growth. The enlarged, relatively monomorphic round, oval, or elongated nuclei are vesicular with prominent nucleoli. The cytoplasm is scant or moderate, more or less intense eosinophilic, and can be rhabdoid in the epithelioid population. Round-cell morphology and uncommonly pleomorphism and hyperchromasia are reported. Real rhabdomyoblasts are not always present. There is a variable, sometimes prominent stromal reaction with sclerosing/hyalinized areas. Immunohistochemically, tumors were all positive with desmin and MYOD1 and to a much lesser degree with myogenin. ALK seems to be heterogeneously expressed in a large subset of cases, and broad-spectrum keratins are positive in almost all cases. S100 can be expressed without concomitant positivity for SOX10 [26,172,174,175]. Differential diagnoses are listed in Table 13.

Most of the tumors behave extremely aggressively, with a reported median survival of 8 months [26,172,173]. However, few patients with local disease and long-term follow-up showed no evidence of disease after treatment, with the mandible being a site of favorable prognosis [172,174,175,176].

## 11. Retroperitoneal Leiomyoma

The first cytogenetic analyzed retroperitoneal leiomyoma harbored a t(10;17)(q22;q21) translocation resulting in a *KAT6B-KANSL1* fusion gene, and the second case was identified with a t(9;22)(q33;q12) leading to an *EWSR1-PBX3* chimeric transcript. Both lesions occurred in woman 45 and 26 years old and showed a usual leiomyoma morphology, with leiomyocytes arranged in long fascicles and a classical immunoprofile with expression of smooth muscle markers. It is obvious that molecular heterogeneity may exist in these tumors [177,178].

## 12. Simple (Unicameral) Bone Cyst (SBC)

SBC was initially reported by Rudolf Virchow in 1876 [179,180,181]. It is a benign intramedullary cystic lesion involving the long bones in skeletally immature individuals. Boys are twice as more affected than girls. The reported peak is between the ages of 3 and 14 years. It commonly arises in the proximal humerus or proximal femur and less frequently in other long bones. Symptoms can be pain and swelling. A unilocular expansile cyst showing double-density fluid levels is radiologically characteristic [5,179,180,182].

**Figure 15:**
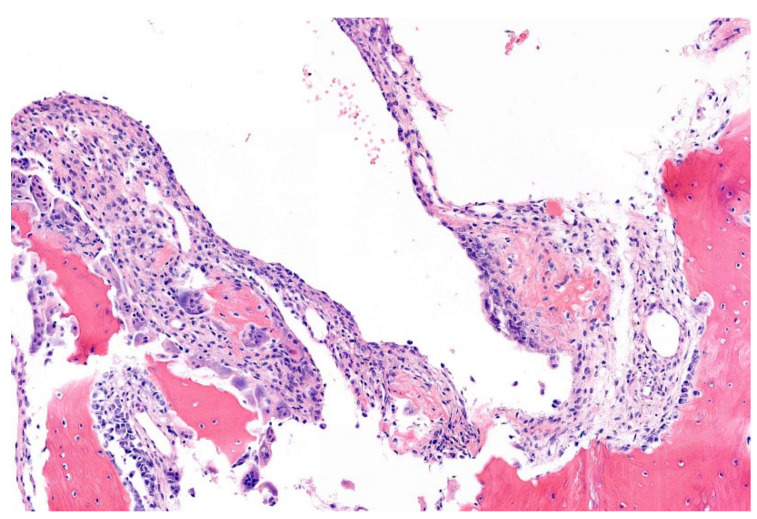
Pseudocystic space of a simple bone cysts lined by myofibroblastic cells. There is primitive osteoid. The preexistent bone shows resorption (HE; 20× magnification).

Macroscopically, an often-fragmented thin fibrous membrane representing the cyst wall possibly with thickening and hemorrhage after trauma is seen (fracture). The fibrous pseudocystic structure is also microscopically obvious showing focal fibrin-like collagen with calcification and ossification. There is no true lining (Figure 15). Myofibroblastic cells and osteoclasts are not as prominent as typically seen in aneurysmal bone cyst (ABC), which is a differential diagnosis. Secondary changes as hemorrhage with resorption with chronic inflammation can be found after fracture. This can camouflage the classical microscopical features [5,179,180,182]. In 2002, the translocation (16;20)(p11.2;q13) was identified as the sole cytogenetic abnormality in a SBC case [183]. Recently, the corresponding *FUS1-NFATC2* or alternatively *EWSR1-NFATC2* fusion have been demonstrated in a subset of cases proving that SBC is a neoplasm [5,180,182] (WHO, Pizem et al., 2020, Hung et al., 2021).

Besides ABC, other differential diagnoses are cystic fibrous dysplasia, intraosseous ganglion, or lipoma (Table 14) [5,180].

Conservative and surgical treatment are discussed in the literature. The recurrence rate is low but fractures are a known complication [5,179,180].

## 13. Hemangioma of Bone with an EWSR1-NFATC1 Fusion

In 1982, Mulliken and Glowacki separated vascular anomalies into hemangiomas and vascular malformations based on their clinicopathological appearance and biological background [184,185]. This was the base for the classification of the International Society for the Study of Vascular Anomalies (ISSVA) [186]. Vascular tumors arise by clonal cellular proliferation of vessels showing a disproportionate growth. In contrast, vascular malformations originate in utero as a result of mosaic mutations leading to erroneous development of vessels with proportionate growth [187].

Hemangiomas of the bone are relatively common and often incidental findings. They can be found at any age and arise often multifocal. The spine is the predilection site followed by the craniofacial bones. Radiologically, well-demarcated lucent lesions with coarse primary trabeculations are visible. When symptomatic, surgical intervention can be necessary and tissue will be obtained [5].

Macroscopically, the lesional tissue, often piecemeal, is soft and hemorrhagic with some bony fragments.

**Figure 16:**
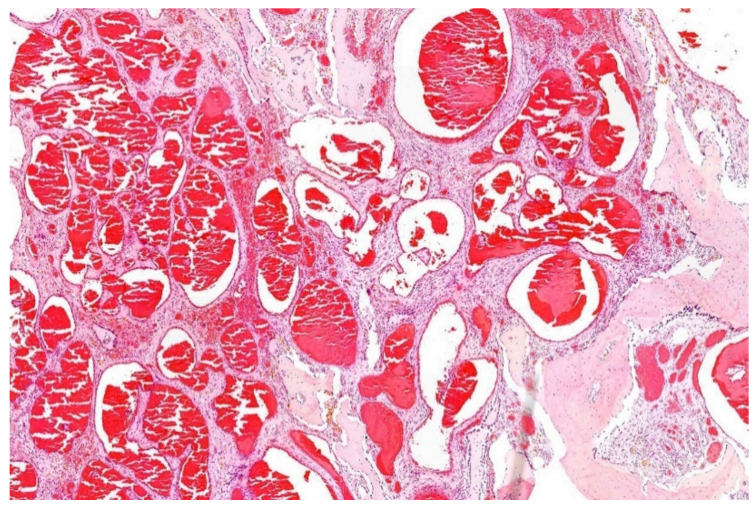
Hemangioma of bone consisting of cavernous blood-filled vessels. Note the surrounding cellular matrix and remodeling of the preexisting bone (HE; 20× magnification).

Microscopy is variable, showing thin and thick-walled vessels of different caliber. The endothelial lining is not remarkable [5]. A stromal reaction can be prominent. A vascular malformation can be difficult to differentiate (Figure 16).

Recently, a multifocal hemangioma has been described located in the occipital bone and clavicle showing a t(18;22)(q23;q12) with an *EWSR1-NFATC1* fusion chimera [188].

Comparable lesions were found with *EWSR1-NFATC1* or *EWSR1-NFATC2* (own observation). Therefore, it seems to be a recurrent finding.

Prognosis is excellent [5,188].

## 14. Conclusions

Several malignant and benign tumors harbor an *EWSR1* rearrangement due to the central role of *EWSR1* in different cell processes and vulnerability of the gene as consequence of frequent transcription. It was the first described fusion gene in sarcomas, and during the last decade(s) its promiscuous character has been shown by involvement in a variety of tumors. The recently described polyphenotypic sarcomas, which seem to be different entities with aggressive behavior, are interesting in this context. Whether they could be treated like Ewing sarcoma or require a more tailored approach is paramount to investigate.

Another intriguing point is that the same fusion partners are present in benign and malignant tumors, arguing that secondary genetic and epigenetic changes are mandatory to regulate and activate the required oncogenic pathways.

## Figures and Tables

**Table 1:** Differential diagnoses of Ewing sarcoma.

Entity	Morphology	IHC	Common Genetic Alterations
CIC-sarcoma	Sheets of undifferentiated round/spindle/epitheloid cells; mild nuclear pleomorphism; and necrosis	CD99 (mostly patchy), WT1, ETV4, DUX4, and NUT (*CIC-NUTM1*)	*CIC-DUX4/FOXO4/LEUTX/NUTM1/2A* fusions
BCOR-sarcoma	Sheets/nests/short fascicles of uniform; bland round-oval-spindle cells; rich capillary network; and myxoid matrix (variable)	BCOR, SATB2, cyclin D1, TLE1, CCNB3 (*BCOR-CCNB3*), and CD99 (50%)	*BCOR-CCNB3/MAML3/ZC3H7B, KMT2D*); *BCOR* ITD*; and *YWHAE-NUTM2B;* *ITD, internal tandem duplication
*EWSR1*-nonETS round-cell sarcomas	Cords/nests/pseudoacinar pattern of round-spindle cells; bland-pleomorphic spectrum; and fibro-/myxohyaline stroma	CD99, NKX2.2, and CKAE1/3 (focal, dot-like)	*EWSR1*/FUS-NFATc2
	Diverse morphology: round-spindle cells; fibrous stroma	Co-expression of myogenic markers (desmin/myogenin/MyoD1), neurogenic markers (S100/SOX10/MITF/GFAP) and keratins (AE1/3)	*EWSR1-PATZ1 or EWSR1-VEZF1*
Desmoplastic small round-cell tumor	Sheets/nests/cords of uniform; bland round cells; and desmoplastic stroma	Desmin (dot-like), keratin, EMA, and WT1 (C-terminus)	*EWSR1-WT1*
Lymphoblastic lymphoma	Small-medium blastoid cells; minimal cytoplasm	CD99, TdT, CD45, CD34, CD1a, and B- and T-cel markers	Diverse
Small-cell carcinoma	Small-medium round-oval cells; salt and pepper chromatin; indistinct nucleoli; molding; and apoptosis	Keratins, CD56, synaptophysin, chromogranin, and TTF1	Diverse; *TP53, PTEN* mutations; *RB1*, 3p loss; and *MYC* amplification
NUT carcinoma	Poorly cohesive sheets of primitive/basaloid cells; abrupt keratinization; and coagulative necrosis	CK5/6, P40, P63, and NUT	*NUT-BRD3/BRD4/NSD3/CIC/BCORL1/MGA/MXD4*
Myoepithelial carcinoma	Solid sheets/nests of cell with high nuclear grade or undifferentiated round-cell morphology; facultatively glandular component; necrosis; and high mitotic count	Pankeratins, S100, EMA, GFAP, SOX10, P63, SMA, calponin, desmin (focal); and INI1 loss (subset)	*EWSR1* rearrangements (various fusion partners); *PLAG1* rearrangements (mixed tumors)
ARMS	Nests with central discohesion-solid nests; monomorphic primitive round cells; and multinucleated wreath-like giant cells	Desmin, myogenin (strong, diffuse), MyoD1, keratin, neuro-endocrine markers (CD56, synaptophysin, and chromogranin)	*PAX3/PAX7-FOXO1*
Sinonasal glomangiopericytoma	Solid-fascicular pattern; spindle-round cells with minimal atypia; arranged around staghorn vessels; and perivascular hyalinization	Beta-catenin (nuclear), SMA	*CTNNB1* mutations
Glomus tumor	Solid-nested pattern; small, uniform round cells with round nucleus, amphophilic-slightly eosinophilic cytoplasm and sharply defined cell borders; and variable vascular pattern	SMA with membranous accentuation, caldesmon, and collagen IV	*MIR143-NOTCH1/2/3*, and *BRAF/KRAS* mutations
Rhabdoid tumor	Solid pattern; rounded-polygonal cells with vesicular nuclei and prominent nucleoli; and eosinophil hyaline-like cytoplasmic inclusions	Diverse; keratins, EMA, CD99, synaptophysin, SALL4, glypican-3, and INI1 loss	*SMARCB1* biallelic loss, *SMARCB1* or *SMARCA4* (germline) mutations
Mesenchymal chondrosarcoma	Biphasic: poorly differentiated round cells and islands of hyaline cartilage; staghorn-like vessels	S100, CD99, SOX9, EMA, desmin, myogenin, and MyoD1	*HEY1-NCOA2*
Synovial sarcoma with round-cell features	Fascicles or sheets of small round hyperchromatic cells; high N/C ratio; staghorn vessels; necrosis; and thin fibrovascular septa	CD99, BCL2, CD56, TLE1, S100 (focal), EMA, and keratins (variable)	*SS18-SSX1/2/4*

**Table 2:** Differential diagnoses of *EWSR1*—non-ETS (*NFATC2* and *PATZ*) fusions.

Diagnosis	Morphology	IHC	Common Genetic Alterations
Solitary fibrous tumor	Patternless pattern of spindle cells or round cells; hyalinized stroma; collagen bundles; staghorn vessels; and possibly fat component	CD34, BCL2, CD99, and STAT6	*NAB2-STAT6*
Myoepithelial tumor	Divers spectrum; reticular/trabecular pattern; variable spindle/epithelioid/plasmocytoid/clear cells; rarely ductal component (mixed tumors); and myxoid stroma	Pankeratins, S100, SOX10, EMA, GFAP, P63, SMA, calponin, and desmin (focal)	*EWSR1* rearrangements (various fusion partners); *PLAG1* rearrangements (mixed tumors)
DSRCT	Sheets/nests/cords of uniform, bland, round cells; and desmoplastic stroma	Desmin (dot-like), keratin, EMA, and WT1 (C-terminus)	*EWSR1-WT1*
CIC-sarcoma	Sheets of undifferentiated round/spindle/epitheloid cells; mild nuclear pleomorphism; and necrosis	CD99 (mostly patchy), WT1, ETV4, DUX4, and NUT (*CIC-NUTM1*)	*CIC-DUX4/FOXO4/LEUTX/NUTM1/2A*
BCOR-sarcoma	Sheets/nests/short fascicles of uniform, bland round-oval-spindle cells; rich capillary network; and myxoid matrix (variable)	BCOR, SATB2, cyclin D1, TLE1, CCNB3 (*BCOR-CCNB3*), and CD99 (50%)	*BCOR-CCNB3/MAML3/ZC3H7B, KMT2D*); *BCOR* ITD; and *YWHAE-NUTM2B*
ARMS	Nests/sheets with central discohesion (pseudoalveolar) or solid nests; monomorphic, primitive round cells; and multinucleated wreath-like giant cells	Desmin, myogenin (strong, diffuse), MyoD1, keratin, and neuro-endocrine markers (CD56, synaptophysin, chromogranin)	*PAX3/PAX7-FOXO1*
Malignant peripheral nerve sheath tumor/Triton	Fascicles of spindle cells and/or sheets epithelioid cells with perivascular accentuation and alternating cellularity; staghorn vessels; geographic necrosis; and heterologous differentiation (rhabdomyoblasts, glandular structures)	S100, SOX10 (focally), and loss of H3K27me3	Inactivating mutations of *NF1*, *CDKN2A/B*, *EED*, and *SUZ2*
Synovial sarcoma	Sheets-fascicles; cellular, monomorphic spindle cells; high N/C ratio; variable epithelial differentiation; staghorn vessels; variable amount of collagen; mast cells; and calcification/ossification; poorly differentiated areas may show round-epithelioid cells	CD99, BCL2, CD56, TLE1, S100 (focal), EMA, and keratins (variable)	*SS18-SSX1/2/4*

**Table 3:** Differential diagnoses of DSRCT (except small blue round-cell sarcoma; see Ewing).

Diagnosis	Morphology	IHC	Common Genetic Alterations
Neuroblastoma	Primitive cells; rosettes; neuropil; and ganglion cells	CD56, synaptophysin, and chromogranin	*nMYC* amplification; *ATRK*, *ALK* mutations; and chromosomal aberrations (1p, 17q, and 11q)
Lymphoma	Variably sized hyperchromatic-blastoid cells with variable atypia; minimal cytoplasm	CD45, B/T-cell markers	Diverse
Blastemic Wilms	Primitive, undifferentiated round-to-spindled cells	WT1 (N-terminus), CD56	*WT1, TP53* mutations; 11p15.5 deletion
Small cell/neuroendocrine carcinoma	Small-medium round-oval cells; salt and pepper chromatin; indistinct nucleoli; molding; and apoptosis	Keratins CD56, synaptophysin, chromogranin, and TTF1	Diverse; *TP53, PTEN* mutations; *RB1*, 3p loss; and *MYC* amplification
Metastatic Merkel cell carcinoma	Round-oval nuclei; high N/C-ratio; salt and pepper chromatin; indistinct nucleoli; molding; and apoptosis	Broad spectrum keratins; CK20 (dot-like), CD56, chromogranin, and synaptophysin	Diverse; depends on polyomavirus (PyV) status; PyV- tumors: *Rb1, TP53* mutations
Small cell mesothelioma	Solid nests; high N/C-ratio; well defined membrane; fine chromatin; and indistinct nucleoli	Calretinin, CK5/6, WT1 (N-terminus), and D2-40	Diverse; *P16* loss; *BAP1* mutation; *NF2* deletion; and *ALK/EWSR1/FUS* rearrangments

**Table 4:** Differential diagnoses of MLS.

Diagnosis	Morphology	IHC	Common Genetic Alterations
Lipoblastoma	Lobulated architecture with fibrous septa with often prominent vasculature; possibly myxoid stroma with possibly plexiform vasculature; resembling fetal fat with prelipoblasts, lipoblasts, and mature fat in variable portions	Not specific	*PLAG1* rearrangements (various fusion partners)
Myxoid pleomorphic liposarcoma	Progressive transition between areas resembling myxoid liposarcoma and pleomorphic liposarcoma; pleomorphic cells; and myxoid matrix	Not specific	*RB1* deletion, *TP53* mutations
Chondroid lipoma	Myxohyaline chondroid matrix; lipoblasts intermingled with mature adipocytes and chondroid cells; and vascularized septa	S100 (mature adipocytes and lipoblasts); keratins (rare)	*C110r95-MRTFB*
Soft tissue angiofibroma	Myxoid-collagenous stroma; prominent, branching vasculature; and bland spindle cells, possibly adipocytes	CD34, EMA, desmine (dendritic cells)	*NCOA2* rearrangements (various fusion partners)
Small, blue round-cell tumors (when round-cell liposarcoma)	Cells with small round-oval-spindle cells with little cytoplasm	See Table Ewing and Ewing-like sarcomas	See Table Ewing and Ewing-like sarcomas

**Table 5:** Differential diagnoses of AFH.

Diagnosis	Morphology	IHC	Common Genetic Alterations
Histiocytic lesions	Diverse; histiocytes; and multinucleated giant cells	Diverse (depending on entity); CD68, CD163, and FXIIIA	Diverse (depending on entity); activating MAPK signaling mutations (*BRAF*, *NRAS*, *KRAS*, *ARAF*, and *MAP2K1*)
Follicular dentritic sarcoma	Fascicles/whorls/storiform pattern; oval-spindle cells with small nucleoli and syncytial borders; nuclear pseudoinclusions; binucleate (often) or multinucleate (rare) forms; and admixed lymphocytic infiltrate with perivascular lymphocytic cuffs	CD21, CD23, CD35, and D2-40	Highly diverse mutational profile
Small, blue round-cell tumors See Table 1 and Table 2	Cells with small round-oval-spindle cells with scant cytoplasm	Diverse (depending on entity)	Diverse (depending on entity)
Epithelioid fibrous histiocytoma	Polypoid; epidermal collarette; epithelioid cells with vesicular nuclei, small nucleoli, and amphophilic cytoplasm	FXIIIA, EMA, and ALK	*ALK* rearrangements (various fusion partners)
Aneurysmatic fibrous histiocytoma	Epidermal hyperplasia and basal layer pigmentation; circumscribed, dermal based proliferation; haphazard arrangement of ovoid spindle cells; admixed foam and giant cell; central blood-filled cystic space; and abundant hemosiderin deposition	FXIIIA, SMA	Not specific
Rhabdomyosarcoma	Monomorphic primitive round cells with variable rhabdomyoblastic differentiation (depending on subtype)	Desmin, myogenin, and MyoD1	Diverse (depending on subtype); *PAX3/PAX7-FOXO1* fusions (ARMS); alterations of RAS signaling pathway (embryonal RMS)
Rhabdoid tumor	Solid pattern; rounded-polygonal cells with vesicular nuclei and prominent nucleoli; and eosinophil hyaline-like cytoplasmic inclusions	Diverse; keratins, EMA, CD99, synaptophysin, SALL4, glypican-3, and INI1 loss	*SMARCB1* biallelic loss
Inflammatory myofibroblastic tumor	Fascicular pattern (variable); plump-spindle cells with vesicular nuclei and small nucleoli; amphophilic cytoplasm; oedematous-myxoid-fibrous stroma; and mixed inflammatory infiltrate	SMA, calponin, desmin, keratin (focal), ALK, and ROS1	*ALK* rearrangements (various fusion partners); *ROS1*, *NTRK3*, *RET*, or *PDGFRB* rearrangements
Carcinoma	Sheets/nests/trabecula; round-oval-epithelioid cells with nuclear atypia and variable amount of cytoplasm	Pankeratins, lineage specific markers (depending on site of origine)	Diverse (depending on site of origin)
Meningeoma	Highly diverse (according to subtype and grade): lobulated; whorled, fascicular pattern; spindle or plump syncytial cells; intranuclear pseudoinclusions; and psammoma bodies	EMA, S100, and PR	Monosomy 22; *NF2* deletions
Extraskeletal myxoid chondrosarcoma	Multinodular; lace-like/reticular pattern; round-spindle monomorphic cells; eosinophilic cytoplasm; and myxoid matrix	Non-specific; S100 (focal)	*NR4A3-EWSR1-/TAF15/TCF12/TFG*
Myoepithelioma (syncytial)	Cutaneous; poorly marginated; syncytial growth; sheets of uniform ovoid-histiocytoid-epithelioid cells; and minimal stroma	S100, EMA, GFAP, SMA, and calponin	*EWSR1-PBX3*
Myxoid liposarcoma	Lobulated; primitive uniform round-ovoid cells; variable number of lipoblasts; myxoid stroma; and plexiform vasculature (chicken wire)	DDIT3	*FUS/EWSR1-DDIT3*

**Table 6:** Differential diagnoses of PPMS.

Diagnosis	Morphology	IHC	Common Genetic Alterations
Salivary gland myoepithelial tumors	Strands/nests/ductular structures of epithelial/myoepithelial cells; and chondromyxoid/hyalinised stroma	Epithelial cells: EMA, cytokeratins; Myoepithelial cells: GFAP, S100, SOX10, p40, p63, and SMA	*PLAG1* or *HMGA2* fusions
Angiomatoid fibrous histiocytoma	Syncytial/whorling pattern (classic AFH); reticular/lace-like pattern (myxoid AFH); uniform histiocytoid cells; blood-filled pseudocysts; and inflammatory/lymphocytic reaction (lymph node-like)	Desmin, EMA, and ALK	*EWSR1-ATF1* or *EWSR1-CREB1*
Extraskeletal myxoid chondrosarcoma	Multinodular; lace-like/reticular pattern; round-spindle monomorphic cells with eosinophilic cytoplasm; and myxoid stroma	Non-specific; S100 (focal)	*NR4A3-EWSR1/TAF15/TCF12/TFG*

**Table 7:** Differential diagnoses of CCS.

Diagnosis	Morphology	IHC	Common Genetic Alterations
Melanoma	Diverse growth patterns; large, atypical spindle-epithelioid-bizarre cells with vesicular nuclei and prominent, eosinophilic nucleoli; nuclear pseudo-inclusions; abundant eosinophilic-clear cytoplasm; and melanin pigment	S100, SOX10, Melan-A, HMB45, and MITF	Diverse: *ARID2*, *BAP1*, *BRAF*, *GNAQ*, *HRAS*, *KIT*, *NF1*, *NRAS*, *PTEN* mutations; and chromosomal gains/losses
Epithelioid Schwannoma	Multilobulated growth; encapsulated; nests or single cells; variableschwannoma epithelioid cells; and myxoid-hyalinized stroma	S100, SOX10, Loss of INI1 (~40%)	Loss of *SMARCB1* (~40%)
Myoepithelial tumors	Divers spectrum; reticular/trabecular pattern; variable spindle/epithelioid/clear/plasmocytoid/rhabdoid cells; rarely ductal component (mixed tumors); fibromyxoid stroma; and hyalinization	Pankeratins, S100, SOX10, EMA, GFAP, P63, SMA, calponin, and desmin (focal)	*EWSR1* rearrangements (various fusion partners); *PLAG1* rearrangements (mixed tumors)

**Table 8:** Differential diagnoses of mesothelioma.

Diagnosis	Morphology	IHC	Common Genetic Alterations
Clear-cell sarcoma	Nested-fascicular pattern; epithelioid-plump spindle cells with vesicular nuclei and macronucleoli; fibrous septa; and scattered wreath-like multinucleated giant cells	Melanocytic markers (S100, SOX10, Melan-A, HMB45, and MITF)	*EWSR1-ATF1/CREB* (most often); *EWSR1-CREM* (rare)
Desmoplastic small round-cell tumor	Sheets/nests/cords of uniform, small round cells; and variable desmoplastic stroma	Desmin (dot-like), keratin, EMA, and WT1 (C-terminus)	*EWSR1-WT1*
Carcinoma	Sheets/nests/trabecules; round-oval-epithelioid cells with nuclear atypia and variable cytoplasm	Pankeratins, lineage-specific markers (depending on site of origin)	Diverse (depending on site of origin)

**Table 9:** Differential diagnoses of myoepithelial tumors.

Diagnosis	Morphology	IHC	Common Genetic Alterations
Extraskeletal myxoid chondrosarcoma	Multinodular; lace-like/reticular pattern; round-spindle monomorphic cells with eosinophilic cytoplasm; and myxoid stroma	Non-specific; S100 (focal)	*NR4A3-EWSR1/TAF15/TCF12/TFG*
Chordoma	Lobulated; fibrous septa; cords/nests of large epithelioid/polygonal cells, physaliphorous cells (bubbly cytoplasm); and variable myxoid stroma	Cytokeratin, EMA, S100, Brachyury	Germline tandem duplication of *TBXT* (rare); germline loss-of-function mutations of *TSC1/2* (rare)
Sclerosing epitheloid fibrosarcoma	Infiltrative; cords/nests of monomorphic epithelioid cells; and hyalinized/sclerotic/collagenous stroma;	MUC4, SMA, and EMA	*EWSR1/FUS/PAX5-CREB3L1/CREB3L2/CREB3L3/CREM*
(Adeno)carcinoma	Sheets/nests/trabeculae, glands; round-oval-epithelioid cells with nuclear atypia and variable cytoplasm; and ductular structures (adenocarcinoma)	Pankeratins, lineage specific markers (depending on site of origin)	Diverse (depending on site of origin)
Small, blue, round cell tumors	Cells with small round-oval-spindle cells with little cytoplasm	Diverse (depending on entity)	See Table 1, Table 2 and Table 3
Epitheloid sarcoma	Nodules of uniform epithelioid-spindled cells with eosinophilic cytoplasm; central geographic necrosis (classic type); multinodular/sheet-like growth; large-slightly pleomorphic epithelioid cells with eosinophilic cytoplasm (proximal type)	CD34, keratins, EMA, and loss of INI1	Loss of *SMARCB1*
Melanoma	Diverse growth patterns; large, atypical spindle-epithelioid-bizarre cells with vesicular nuclei and prominent, eosinophilic nucleoli; nuclear pseudo-inclusions; abundant eosinophilic-clear cytoplasm; and melanin pigment	S100, SOX10, Melan-A, HMB45, and MITF	Diverse: *ARID2*, *BAP1*, *BRAF*, *GNAQ*, *HRAS*, *KIT*, *NF1*, *NRAS*, *PTEN* mutations; and chromosomal gains/losses
Epithelioid schwannoma	Multilobulated growth; capsule; nests or singly cells; variable epithelioid cells; and myxoid-hyalinized stroma	S100, SOX10, and loss of INI1 (~40%)	Loss of *SMARCB1* (~40%)
Epitheloid malignant peripheral nerve sheath tumor	Lobulated growth; atypical epithelioid cells with enlarged nuclei; and prominent nucleoli and eosinophilic cytoplasm	S100, SOX10 (strong and diffuse), and loss in INI1 (~75%)	Loss of *SMARCB1* (~75%)
Ossifying fibromyxoid tumor (mostly benign, rarely malignant)	Multilobulated; nests/cords of uniform round-spindle cells; indistinct cytoplasm; no atypia (rarely high nuclear grade in malignant lesions); fibromyxoid stroma; partial rim of mature bone; and atypical osteoid in malignant tumors	S100, desmin, GFAP (focal), and pankeratin (rare)	*PHF1* rearrangements (diverse fusion partners)
Clear-cell sarcoma	Nested-fascicular pattern; epithelioid-plump spindle cells with vesicular nuclei and macronucleoli; fibrous septa; and scattered wreath-like multinucleated giant cells	Melanocytic markers (S100, SOX10, Melan-A, HMB45, and MITF)	*EWSR1-ATF1/CREB* (most often); *EWSR1-CREM* (rare)
Malignant rhabdoid tumors	Solid pattern; uniform rounded-polygonal cells with vesicular nuclei and prominent nucleoli; and eosinophilic hyaline-like cytoplasmic inclusions	Diverse; keratins, EMA, CD99, synaptophysin, SALL4, glypican-3, and INI1 loss	*SMARCB1* biallelic loss
Epithelioid hemangio-endothelioma	Infiltrative, sometimes angiocentric growth; cords/nests of bland looking epithelioid and spindle cells; glassy cytoplasm; intracytoplasmic vacuoles (blister cells); and myxohyaline stroma	CD34, CD31, ERG, D2-40, keratins (subset), SMA, CAMTA1, and TFE3	*WWTR1-CAMTA1* (>90%); *YAP1-TFE3*
Pseudomyogenic (epitheloid sarcoma-like) hemangio-endothelioma	Multiple discontinuous nodules; possibly involvement of different tissue planes; sheets/fascicles of plump-spindle-epithelioid cells with abundant, brightly eosinophilic cytoplasm; vesicular nuclei with small nucleoli; mild nuclear atypia; not obvious vascular; and prominent stromal neutrophils (50%)	Keratins (AE1/AE3 but not MNF116), FLI, ERG, CD31 (50%), SMA (focal), and FOSB	*SERPINE1/ACTB-FOSB*

**Table 10:** Differential diagnoses of LGFMS.

Diagnosis	Morphology	IHC	Common Genetic Alterations
Desmoid-type fibromatosis	Long, sweeping fascicles; slender uniform spindle cells; pale cytoplasm; and parallel to fascicles thin-walled blood vessels with perivascular edema	Beta-catenin (nuclear), SMA, and desmin (focal)	*CTNNB1* or *APC* mutations
Nodular fasciitis	Plump spindle cells; tissue-culture aspect; extravasated erythrocytes; lymphocytes; and sometimes osteoclast-like giant cells	Non-specific: SMA, CD68, and desmin (focal)	*USP6* rearrangements (diverse fusion partners)
Ossifying fibromyxoid tumor (mostly benign, rarely malignant)	Multilobulated; nests/cords of uniform round-spindle cells; indistinct cytoplasm; no atypia (rarely high nuclear grade in malignant lesions); fibromyxoid stroma; partial rim of mature bone; and atypical osteoid in malignant tumors	S100, desmin, GFAP (focal), and pankeratin (rare)	*PHF1* rearrangements (diverse fusion partners)
Neurofibroma	Nodular or diffuse growth; variable admixture of perineurial cells, schwann cells and fibroblasts; hyperchromasia; usually no atypia or mitoses; and myxoid-collagenous stroma with “shredded-carrot” collagen	S100, SOX10, CD34, and EMA	*NF1* deletions
Perineurioma	Nodular; non-encapsulated; circumscribed or infiltrative; whorled/storiform/fascicular pattern; and slender spindle cells with bipolar cytoplasmic extensions and uniform oval or elongated nuclei	EMA, GLUT1, CD34, and Claudin 1	*TRAF7* mutations (intraneural perineurioma); *NF1/2* deletions
Desmoplastic fibroblastoma	Paucicellular; bland stellate-spindle cells; and abundant collagenous-myxocollagenous stroma	FOSL1, SMA (focal)	t(2;11)
Malignant peripheral nerve sheath tumor	Fascicles of monomorphic atypical spindle cells with perivascular accentuation and alternating cellularity; pleomorphism is possible; staghorn vessels; geographic necrosis; and heterologous differentiation	S100, SOX10 (focal), and loss of H3K27me3	Inactivating mutations of *NF1*, *CDKN2A/B*, *EED*, and *SUZ2*
Fibroma nuchae	Paucicellular; bland spindle cells; thick collagen bundles; and entrapped adipose tissue and/or small nerves	CD34	Not relevant
Intramuscular (cellular) myxoma	Myxoid stroma; hypocellular; uniform spindle-stellate cells; inconspicuous vessels; and infiltration into skeletal muscle	CD34	*GNAS* mutations
Dermatofibrosarcoma protuberans	Dermal based; cellular, storiform pattern of uniform spindle cells; encasement of skin appendages; and infiltration in subcutaneous fat with honeycombing	CD34	*COL1A1-PDGFB* (most often); *COL6A3-PDGFD* or *EMILIN2-PDGFD* (rare)
*NTRK*-rearranged spindle cell neoplasm (emerging)	Wide spectrum of morphologies and histological grades; most often haphazardly arranged monomorphic spindle cells; variable stromal/perivascular hyalinization; and infiltrative growth into fat	S100, CD34 (co-expression), and NTRK	*NTRK1-3* rearrangements (diverse fusion partners); *RAF1* or *BRAF* fusions (rare)

**Table 11:** Differential diagnoses of SEF.

Diagnosis	Morphology	IHC	Common Genetic Alterations
Ossifying fibromyxoid tumor	Multilobulated; nests/cords of uniform round-spindle cells; indistinct cytoplasm; no atypia (rarely high nuclear grade in malignant lesions); fibromyxoid stroma; partial rim of mature bone; and atypical osteoid in malignant tumors	S100, desmin, GFAP (focal), and pankeratin (rare)	*PHF1* rearrangements (diverse fusion partners)
Carcinoma (lobular, signet ring cell)	Files-small nests; round-oval cells with variable cytoplasm and nuclear atypia; and intracytoplasmic mucin vacuole	Pankeratins, lineage specific markers (depending on site of origine)	Diverse (depending on site of origin)
Sclerosing lymphoma	Variably sized hyperchromatic-blastoid cells with variable atypia; scant cytoplasm; and sclerotic stroma	CD45, B/T-cell markers	Diverse
Synovial sarcoma	Sheets-fascicles; cellular, monomorphic spindle cells; high N/C ratio, variable epithelial differentiation; staghorn vessels; variable amount of collagen; mast cells; and calcification/ossification; poorly differentiated areas may show round-epithelioid cells	CD99, BCL2, CD56, TLE1, S100 (focal), EMA, and keratins (variable)	*SS18-SSX1/2/4*
Clear-cell sarcoma	Nested-fascicular pattern; epithelioid-plump spindle cells with vesicular nuclei and macronucleoli; fibrous septa; and scattered wreath-like multinucleated giant cells	Melanocytic markers (S100, SOX10, Melan-A, HMB45, and MITF)	*EWSR1-ATF1/CREB* (most often); *EWSR1-CREM* (rare)
Osteosarcoma	Highly diverse; infiltrative growth; severely anaplastic and pleiomorphic cells; monomorphic small cell appearance is rare; and atypical neoplastic bone formation (essential)	SATB2, osteocalcin (*BGLAP*), osteonectin (*SPARC*), osteoprotegerin (*TNFRSF11B*), RUNX2, S100, actins, CD99, keratin, and EMA	Highly complex chromosomal aneuploidy
Myoepithelioma	Divers spectrum; reticular/trabecular pattern; variable spindle/epithelioid/clear/plasmocytoid/rhabdoid cells; rarely ductal component (mixed tumors); fibromyxoid stroma; and hyalinization	Pankeratins, S100, EMA, GFAP, SOX10, P63, SMA, calponin, and desmin (focal)	*EWSR1* rearrangements (various fusion partners); *PLAG1* rearrangements (mixed tumors)

**Table 12:** Differential diagnoses of *EWSR1-SMAD3*-positive fibroblastic tumor.

Diagnosis	Morphology	IHC	Common Genetic Alterations
Cellular schwannoma	Encapsulation; predominantly Antoni A areas; variable neuroid spindle cells; possibly hyperchromasia and frequent mitoses; rarely Verocay bodies or schwannian whorls; and hyalinized vessels subcapsular lymphocytes	S100, SOX10	*NF2* deletion; *LATS1/2, ARID1A/1B*, and *DDR1* mutations; *SH3PXD2A-HTRA1*
Perineurioma	Whorled/storiform pattern; slender spindle cell with bipolar cytoplasmic extensions and oval/elongated uniform nuclei	EMA, GLUT1, CD34, Claudin 1	*TRAF7* mutations (intraneural perineurioma); *NF/2* deletions
Myofibroma/myopericytoma	Myofibroma: nodular; biphasic pattern: immature plump-spindle cells around staghorn vessels; and nodules/fascicles of hyalinized-myoid-chondroid appearing cells. Myopericytoma: lobular; variably cellular; bland oval-spindle-myoid cells; and perivascular growth	SMA (myofibroma/myopericytoma), caldesmon (myopericytoma)	*PDGFRB*, *NOTCH3* mutations; *SRF-RELA*
Angioleiomyoma	Bundles of bland leiomyocytes around numerous vessels	SMA, calponin, caldesmon, and desmin (variable)	Not relevant
Cellular digital fibroma	Whorls/short fascicles; bland spindle cells with slightly eosinophilic cytoplasm; pale pink-red, paranuclear inclusions; and collagenous stroma	Actin, calponin, and caldesmon	Not relevant
Calcifying aponeurotic fibroma	Fibromatosis-like, infiltrative component of bland spindle cells; nodular calcified component with hyalinized-chondroid matrix encircled by rounded-chondrocyte like cells; and osteoclast-type giant cells	SMA, CD99, S100 (chondroid areas)	*FN1-EGF*
Acral fibromyxoma	Nodular or diffuse; infiltrative growth of bland spindle-stellate cells; and variably myxoid-collagenous stroma	CD34, EMA, SMA, and RB1 (loss)	*RB1* deletions
Superficial fibromatosis	Plexiform or multinodular; moderately cellular bland spindle cells; and collagenous stroma	SMA, desmin, beta-catenin (nuclear) positive in a subset of cases despite absence of *CTNNB1* or *APC* mutation	Not relevant
Cellular dermatofibroma	Radiar configuration; storiform/short fascicular pattern; cellular center of fibrohistiocytic cells; and peripheral collagen entrapment	SMA, FXIIA, and CD68	*PRKCB/PRKCD-PDPN/CD63/LAMTOR1*
Lipofibromatosis	Admixture of mature fat, fascicles of bland myofibroblastic spindle cells (fibromatosis-like) and lipoblast-like cells at the interface	CD34, SMA	*EGFR/HER1/ROS/RET/PDGFRB-EGF/HBEGF/TGF-α*
Smooth muscle neoplams	Intersecting fascicles of smooth muscle cells; blunt-ended, cigar-shaped nuclei; and eosinophilic cytoplasm	SMA, desmin, and caldesmon	Not relevant;
Synovial sarcoma	Sheets-fascicles; cellular, monomorphic spindle cells; high N/C ratio; variable epithelial differentiation; staghorn vessels; variable amount of collagen; mast cells; calcification/ossification; and poorly differentiated areas may show round-epithelioid cells	CD99, BCL2, CD56, TLE1, S100 (focal), EMA, and keratins (variable)	*SS18-SSX1/2/4* fusion
Malignant peripheral nerve sheath tumor	Fascicles of spindle cells with perivascular accentuation and alternating cellularity; staghorn vessels; georgraphic necrosis; and heterologous differentiation	S100, SOX10 (focal), and loss of H3K27me3	Inactivating mutations of *NF1*, *CDKN2A/B*, *EED*, and *SUZ2*
Acral dermatofibrosarcoma protuberans	Dermal-based; cellular, storiform pattern of uniform spindle cells; encasement of skin appendages; and infiltration in subcutaneous fat with honeycombing	CD34	*COL1A1-PDGFB* fusion (most often); *COL6A3-PDGFD* or *EMILIN2-PDGFD* (rare)
Low-grade fibromyxoid sarcoma	Alternating myxoid-fibrous areas; whorling/fascicular pattern of bland spindle cells; arcades of small vessels; and sometimes hyaline rosettes, which sometimes overlap with sclerosing epitheloid fibrosarcoma	MUC4 (highly sensitive and specific); EMA, S100, CD34, and SMA (variable)	*EWSR1/FUS-CREB3L1/2*
Pseudomyogenic (epitheloid sarcoma-like) hemangioendothelioma	Multiple discontinuous nodules; possible involvement of different tissue planes; sheets/fascicles of plump-spindle-epithelioid cells with abundant, brightly eosinophilic cytoplasm; vesicular nuclei with small nucleoli; mild nuclear atypia; not obvious vascular; and prominent stromal neutrophils (50%)	Keratins (AE1/AE3 but not MNF116), FLI, ERG, CD31 (50%), SMA (focal), and FOSB	*SERPINE1/ACTB-FOSB*
NTRK-rearranged spindle cell neoplasms	Wide spectrum of morphologies and histological grades; most often haphazardly arranged monomorphic spindle cells; variable stromal/perivascular hyalinization; and infiltrative growth into fat	S100, CD34 (co-expression), and NTRK	*NTRK1-3* rearrangements (diverse fusion partners); *RAF1* or *BRAF* fusions (rare)

**Table 13:** Differential diagnoses of epithelioid and spindle cell rhabdomyosarcoma.

Entity	Morphology	IHC	Common Genetic Alterations
Inflammatory myofibroblastic tumor	Fascicular pattern (variable); plump-spindle cells with vesicular nuclei and small nucleoli and amphophilic cytoplasm; oedematous-myxoid-fibrous stroma; and mixed inflammatory infiltrate	SMA, calponin, desmin, keratin (focal), ALK, and ROS1	*ALK* rearrangements (various fusion partners); *ROS1*, *NTRK3*, *RET*, or *PDGFRB* rearrangements
Carcinoma	Sheets/nests/trabecules; round-oval-epithelioid cells with variable cytoplasm and nuclear atypia	Pankeratins, lineage-specific markers (depending on site of origine)	Diverse (depending on site of origin)
Myoepithelial carcinoma	Solid sheets/nests of variable myoepithelial cells (epitheloid, spindled, plasmocytoid, rhabdoid, and clear) with high nuclear grade or undifferentiated round-cell morphology; necrosis; and high mitotic count	Pankeratins, EMA, S100, SOX10, GFAP, P63, SMA, calponin, desmin (focal); and INI1 loss (subset)	*EWSR1* rearrangements (various fusion partners); *PLAG1* rearrangements (mixed tumors)
*EWSR1-PATZ1* sarcoma	Diverse morphology: round-spindle cells; fibrous stroma	Co-expression of myogenic markers (desmin/myogenin/MyoD1) and neurogenic markers (S100/SOX10/MITF/GFAP)	*EWSR1-PATZ1*
Dedifferentiated chondrosarcoma	Conventional chondrosarcoma with abrupt transition to a high-grade non-cartilaginous sarcoma (undifferentiated sarcoma, osteosarcoma, angiosarcoma, leiomyosarcoma, and rhabdomyosarcoma)	Diverse (according to line of differentiation); loss of H3K27me3, MDM2 overexpression, p53 overexpression, and PDL1	Complex karyotype; *IDH1/2*, *TP53* mutations
Pseudomyogenic (epitheloid sarcoma-like) hemangioendothelioma	Multiple discontinuous nodules; possibly involvement of different tissue planes; sheets/fascicles of plump-spindle-epithelioid cells with abundant, brightly eosinophilic cytoplasm; vesicular nuclei with small nucleoli; mild nuclear atypia; not obvious vascular; and prominent stromal neutrophils (50%)	Keratins (AE1/AE3 but not MNF116), FLI, ERG, CD31 (50%), SMA (focal), and FOSB	*SERPINE1/ACTB-FOSB*
Rhabdomyosarcoma (spindle cell)	Cellular fascicles with intersecting/herringbone pattern; atypical uniform spindle cells with pale eosinophilic cytoplasm; primitive round cells may be present; and tadpole/strap cells (sometimes)	Desmin, MyoD1 (focal or diffuse), myogenin (focal)	*SRF/VGLL2/TEAD1-NCOA2, VGLL2-CITED2* (congenital spindle cell RMS); and *MYOD1* mutation
Leiomyosarcoma	Intersecting fascicles of smooth muscle cells; blunt-ended, cigar-shaped nuclei; variable atypia and pleomorphism (depending on grade); eosinophilic cytoplasm; mitoses; necrosis	SMA, desmin, and caldesmon	Extensive genomic instability (leiomyosarcoma); diverse gene involvement with p53 mutations; deleterious ATRX alterations; ALK rearrangement (small subset); and *NF1* mutations (subset of inflammatory leiomyosarcoma);
Melanoma	Diverse growth patterns; large, atypical spindle-epithelioid-bizarre cells with vesicular nuclei and prominent, eosinophilic nucleoli; nuclear pseudo-inclusions; abundant eosinophilic-clear cytoplasm; and melanin pigment	S100, SOX10, Melan-A, HMB45, and MITF	Diverse: *ARID2*, *BAP1*, *BRAF*, *GNAQ*, *HRAS*, *KIT*, *NF1*, *NRAS*, and *PTEN* mutations; chromosomal gains/losses
Malignant peripheral nerve sheath tumor	Fascicles of spindle cells with perivascular accentuation and alternating cellularity; staghorn vessels; necrosis; and heterologous differentiation	S100, SOX10 (focal), and loss of H3K27me3	Inactivating mutations of *NF1*, *CDKN2A/B*, *EED*, and *SUZ2*

**Table 14:** Differential diagnoses of simple (unicameral) bone cyst.

Diagnosis	Morphology	Common Genetic Alterations
Fibrous dysplasia	Irregular, curvilinear trabeculae of woven (or rarely lamellar) bone without osteoblast rimming; inconspicuous osteoblasts; sharpey fibers; and osteoclasts bland fibroblastic stroma	*GNAS* mutations (50–70%)
Intraosseous ganglion	Cavity without lining; filled with mucoid viscous material	None (probably degenerative)
Lipoma	Nodules of mature adipose tissue, often fibrotic	Unknown

## Data Availability

Not applicable.
